# hnRNPC regulates cancer-specific alternative cleavage and polyadenylation profiles

**DOI:** 10.1093/nar/gkz461

**Published:** 2019-05-31

**Authors:** Harry Fischl, Jonathan Neve, Zhiqiao Wang, Radhika Patel, Alastair Louey, Bin Tian, Andre Furger

**Affiliations:** 1 Department of Biochemistry, University of Oxford, Oxford OX1 3QU, UK; 2 Department of Microbiology, Biochemistry and Molecular Genetics, Rutgers New Jersey Medical School and Rutgers Cancer Institute of New Jersey, Newark, NJ 07103, USA

## Abstract

Alternative cleavage and polyadenylation (APA) can occur at more than half of all human genes, greatly enhancing the cellular repertoire of mRNA isoforms. As these isoforms can have altered stability, localisation and coding potential, deregulation of APA can disrupt gene expression and this has been linked to many diseases including cancer progression. How APA generates cancer-specific isoform profiles and what their physiological consequences are, however, is largely unclear. Here we use a subcellular fractionation approach to determine the nuclear and cytoplasmic APA profiles of successive stages of colon cancer using a cell line-based model. Using this approach, we show that during cancer progression specific APA profiles are established. We identify that overexpression of hnRNPC has a critical role in the establishment of APA profiles characteristic for metastatic colon cancer cells, by regulating poly(A) site selection in a subset of genes that have been implicated in cancer progression including *MTHFD1L*.

## INTRODUCTION

Cleavage and polyadenylation (CPA) is a fundamental pre-mRNA processing reaction that matures the 3′ end of almost all protein-coding genes. The reaction is executed by the cleavage and polyadenylation complex that recognises the poly(A) signals located in pre-mRNAs. The generic core poly(A) signal consists of a hexamer [A(A/U)UAAA] and a U/GU rich downstream element (DSE). The cleavage and polyadenylation complex assembles on these sequence elements by direct interaction with their multi-subunit components CPSF and CSTF respectively (1). After assembly, the complex catalyses first the cleavage of the pre-mRNA at the poly(A) site and subsequently polyadenylates the 3′ end of the emerging mRNA. Most mammalian genes contain multiple poly(A) signals in their primary transcripts and alternative cleavage and polyadenylation (APA), by analogy to alternative splicing, vastly increases the mRNA repertoire in cells. Depending on the location of the alternate poly(A) sites within the gene, the resulting transcripts can differ in length and their coding potential, forthwith referred to as coding region APA (CR-APA). The second type of transcripts that can be generated by APA code for identical proteins and differ only in the length of their 3′ untranslated regions (UTRs) and are summarised as UTR-APA events. UTR-APA is highly promiscuous, with more than half of expressed human genes generating mRNA isoforms that differ in the sequence composition of their 3′UTRs (2). The physiological impact of CR-APA resides in the scope to encode different proteins. Whilst UTR-APA results in mRNA isoforms that encode the same protein, their 3′ UTRs can differ widely in the number and composition of RNA binding protein (RBP) sites and miRNA target sites. Inclusion or exclusion of such sites by APA provides cells with opportunities to regulate gene expression at the post-transcriptional level by affecting transcript stability, translational output and subcellular localisation (3). UTR-APA therefore has the capacity to regulate final protein output and destination (4). Given the regulatory potential, it is not surprising that in the last decade the regulation of APA has been shown to be critical for cells to achieve adequate gene expression in response to specific cues including stress, proliferation and differentiation; and its deregulation can contribute to disease progression (5,6). Some of the most prominent APA profile changes are observed between normal and cancer cells with a tendency to express mRNA APA isoforms with shorter UTRs in the latter (7–11). This shortening, in some cases, may boost the translational output of key genes such as cell cycle regulators by avoiding exposure to repressive modules such as miRNAs (11). However, despite considerable efforts and analyses of numerous APA profiles from cancer cells, how these characteristic profiles are established and what their physiological consequences are, is largely unknown (12). Elucidating the mechanisms is complicated by the fact that the steady state APA profile within a cell depends not only on which poly(A) sites are selected at the point of cleavage but also on the post-transcriptional fate of each isoform (6).

Recently, subcellular fractionation approaches have been developed that allow better resolution between active and passive APA events (13,14). In addition, as many APA events appear to be restricted to the nucleus (13), for the identification of physiologically relevant APA events, it is important to characterize cytoplasmic APA profiles. Here, we used a colon cancer-based tissue culture cell system combined with a subcellular fractionation approach to identify factors that control specific, physiologically relevant APA events. We identified hnRNPC as a critical regulator for the establishment of cancer characteristic profiles in a subset of genes including *MTHFD1L*, which has been strongly linked to cancer progression. We show that an increase in hnRNPC in metastatic derived colon epithelial cells contributes to the upregulation of full-length *MTHFD1L* mRNA production by controlling poly(A) site choice. Our results thus highlight hnRNPC as a critical regulator of physiologically relevant APA events that may contribute to carcinogenesis by modulating expression of genes that regulate cell proliferation and metastasis. Importantly, we identify similar hnRNPC dependent APA profile shifts in RNA-seq data from patient derived tumour and normal colon epithelial cells.

## MATERIALS AND METHODS

### Description of cell types and cell culture

The 1CT cell line is a non-malignant adult-derived human male colonic epithelial cell line (15). The cell line was immortalized with the non-oncogenic proteins cyclin-dependent kinase 4 (CDK4), allowing the cells to bypass the normal cell culture associated stresses that can lead to senescence, and human telomerase (hTERT), thereby allowing telomeres to be maintained and preventing replicative senescence (15). Characterization of the CDK4 and hTERT expressing immortalized 1CT cell line has shown that ∼98% of cells display the colon epithelial cell specific marker A33 (15). The cancerous cell lines SW480 and SW620 are both derived from the same patient: a 51-year-old Caucasian male. The SW480 cell line was established from a Dukes’ type B primary adenocarcinoma of the colon and the SW620 cell line was derived from a lymph node after cancer recurred with widespread metastasis (16). SW480 and SW620 cells were cultured in Dulbecco's modified Eagle's medium (DMEM) containing 10% (v/v) foetal calf serum (FCS), 2 mM glutamine and Penicillin/Streptomycin (100 mg/ml). Cells were grown to 60–80% confluence before harvesting or passaging unless specified otherwise. 1CT cells were cultured as described previously (15).

### RNAi and western blotting

Scrambled negative control siRNA (ThermoFisher, 4390843) or siRNAs targeting *ELAVL1* (ThermoFisher, s4609) or *hnRNPC* (ThermoFisher, s6721) were transfected in a reaction mix containing Opti-MEM medium (Sigma, 31985062) and Lipofectamine RNAiMAX (ThermoFisher, 13778075) when the SW620 cells reached 30% confluence. siRNA containing media was replaced by normal DMEM media 24 h after the first transfection. Cells were transfected again 24 h after the replacement of the normal media. RNA from nuclear and cytoplasmic subcellular fractions and whole cell protein were extracted 24 h after the second transfection. Efficient knockdown was confirmed by western blotting using hnRNPC (GeneTex, GTX113463) and ELAVL1 (abcam, ab200342) antibodies.

Western blotting was also used to determine the level of MTHFD1L using monoclonal MTHFD1L antibody (SantaCruz, D-7) following hnRNPC siRNA knockdown. An antibody for HSP-60 (Bethyl, A302-845A) was used as a loading control. For comparisons of protein expression between the different cell lines an antibody targeting CARM1 (Bethyl, A300-421A) was used as a loading control.

### Real time quantitative RT-PCR (qRT-PCR)

4 μg of purified cytoplasmic RNA was incubated (37°C, 1 h) with 2 μl DNase I buffer, 1 μl RNaseOUT (Invitrogen), 1 μl DNase I (Roche) in a final volume of 20 μl then heated (70°C, 15 min). 0.5 μg RNA was reverse transcribed by incubating (65°C, 5 min) with 0.6 μl random primers, 1 μl dNTPs in a 13 μl final volume and then adding 4 μl First-Strand buffer, 1 μl 0.1M DTT, 1 μl RNaseOUT, 1 μl Superscript III (replaced with 1 μl H_2_O in control reactions) and incubating at 50°C (1 h) and then 70°C (15 min). Complementary DNA (cDNA) levels relative to genomic DNA standards for short and long transcript isoforms, using primers (Supplementary Figure S21) mapping to upstream of the short and long isoform poly(A) sites, respectively, were established using Real Time quantitative PCR (qPCR) using a RotorGene (Corbett) and SYBR Green mix (Bioline). Barcharts show the mean log_2_ ratio of short to long transcript isoforms averaged across all biological replicates. Error bars show the standard deviation.

### Cell fractionation, RNA isolation, library preparation and sequencing

Cell fractionation was performed as described (13). The Quant-Seq 3′mRNA-Seq kit supplied by Lexogen was used for extraction and deep sequencing of the 3′ ends. The manufacturer's protocol was followed, using 500 ng of input RNA for each sample and 13 cycles of PCR. The resulting libraries were loaded onto the Ion Chef platform for template preparation and the prepared chip was then sequenced on the Ion Proton Sequencing system as per manufacturer's instructions.

Sequences were aligned using the Ion Torrent Server TMAP aligner to genome build hg19. Reads mapping to true poly(A) sites were then extracted by using the Bedtools Intersect function to select only reads that fell within 100 nucleotides of a previously confirmed poly(A) site. These poly(A) sites were those identified using 3′ READS in HEK293 or HBL cells, or those identified using PolyA-Seq in several different human tissues under accession number GSE30198 (17). The resulting selected reads were then analysed using the APA analysis platform as described in (13,18). In brief, CPA sites located within 24 nt as mapped by polyA site (PAS) reads from all samples were clustered. The site with the most PAS reads was selected to represent the CPA site cluster. All the reads in a cluster were used to calculate isoform abundance. CPA sites were mapped to RefSeq transcripts. CPA sites located downstream of RefSeq transcripts were linked to the transcript by cDNA, EST and directional paired-end RNA-seq data from the ENCODE project. An extended region is covered by cDNA/EST sequences or RNA-seq reads without a gap greater than 40 nt. We also required that the 3′UTR extension did not exceed the transcriptional start site or 3′splice site of any other gene on the same strand. The relative abundance of a transcript isoform in a sample was defined as the proportion of PAS reads supporting this isoform in all the PAS reads mapped to the gene. To remove data noise, CPA sites with relative abundance <5% in all of the samples were discarded.

The full polyadenylated RNA-seq was performed with fractionated RNA from 1CT and SW620 cells which was processed with the Ion Total RNA-Seq Kit v2. Sequences were aligned using bowtie to genome build hg19.

Sequencing data were visualized using IGV after normalizing data to reads per million (RPM) and window functions set to ‘Mean’ and ‘Autoscale’.

### Motif analysis

Alternative UTR sequences were extracted, and the Discriminative Regular Expression Motif Elicitation (DREME) motif analysis tool (19) was used with default parameters to identify overrepresented motifs. The reference sequences used were aUTRs from examples which showed the opposite trend to those being analysed. Subsequently, the motifs identified were submitted to the TOMTOM motif comparison tool (20) to identify any RNA-binding proteins that may be binding these motifs. The motif database used for known RNA-binding proteins was generated using the RNAcompete method (21).

### Analysis of tumour and normal tissue-derived RNA-seq samples

Data were obtained from (22) in which RNA from normal and tumour samples from 103 colon cancer patients had been sequenced. For each sample, reads that aligned to windows (Supplementary Figure S18) covering regions of the terminal exon of the short and long isoforms of *MTHFD1L, MAP4, CLCN7, OTUB1, ATP2A2, SMAD3* and *FBRSL1* were counted. A ratio of short/long isoform counts was calculated for each gene for each sample. Bar charts show the mean of these ratios for all normal and all tumour samples. Error bars show the S.E.M. A Student's *t*-test was used to test the significance of the difference between these mean ratios. The number of reads aligning to the terminal exon of *hnRNPC* per million total aligned reads was also calculated for each sample. The bar chart shows the mean for all normal and tumour samples. Error bars show the S.E.M. A Student's *t*-test was used to test the significance of the difference between these means.

## RESULTS

Colorectal cancer cell lines have been shown to be a highly representative cell-based system of primary tumours and suitable for the study of colorectal cancer biology (23). In order to identify factors that control APA during cancer progression we chose three distinct cell lines: the non-malignant adult-derived human male colonic epithelial 1CT cell line (15) and the SW480 and SW620 cell lines, which both derive from the same patient. The SW480 cell line was established from a Dukes’ type B primary adenocarcinoma of the colon and the SW620 cell line was derived from a lymph node after cancer recurred with widespread metastasis (16). Although these cells have undergone long term culture they represent a valid model for studying the comparative levels of tumorigenicity (24). The comparison of the 1CT, SW480 and SW620 cell lines, therefore, provide a rare model in which changes in APA profiles throughout the onset and metastases process of colorectal cancer and their underlying mechanism can be evaluated using the subcellular fractionation approach.

### Cancer progression is associated with distinct nuclear and cytoplasmic APA profiles

To determine the nuclear and cytoplasmic APA profiles of the three cell lines, 1CT, SW480 and SW620 cells were fractionated (Supplementary Figure S1) and RNA fractions isolated as previously described (13). The 3′ regions of the isolated RNA were extracted using the QuantSeq 3′ mRNA-Seq library kit and libraries were processed on the Ion Chef platform and subsequently sequenced on the Ion Proton system. To subtract internal priming events, mapped reads were then filtered (Supplementary Figure S2) retaining those which overlap with confirmed poly(A) sites (13,17) and passed through an established APA analysis pipeline (13,18,25). Nuclear-retained lncRNA *MALAT1* shows a >250-fold enrichment in the nuclear fractions relative to the corresponding cytoplasmic fractions confirming efficient fractionation in all isolations (Supplementary Figure S3). Principal component analysis shows that the biological replicates are highly similar (Supplementary Figure S4) confirming the reproducibility of the approach and highlights that the different cells do display unique gene expression profiles.

**Figure 1.**
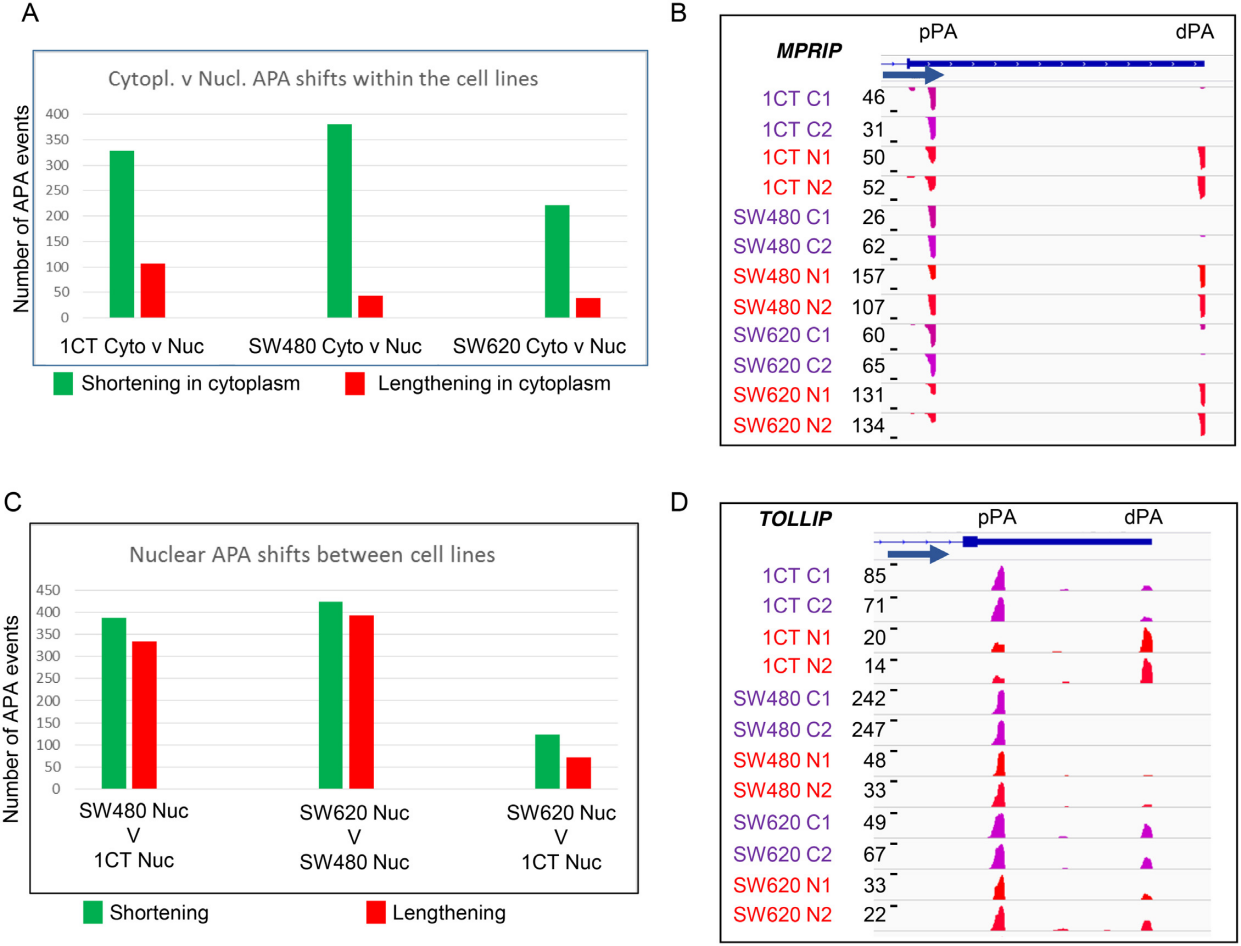
Nuclear and cytoplasmic UTR-APA profile comparison. (A and C) Bar charts displaying the number of events showing significant differential APA isoform representation when comparing UTR-APA isoform abundances. The identity of the samples being compared is shown under each comparison with each bar representing events where the shorter APA isoforms (green) or longer APA isoforms (red) have a significantly higher relative representation in the first sample. Significant differentially represented APA isoforms are those in which *P*≤0.01 (Fisher's exact test) and the isoform abundance change is ≥5% relative to total APA isoforms associated with that gene. (**A**) Shows the number of significant UTR-APA shortening (green) and lengthening (red) events occurring when comparing nuclear to cytoplasmic fractions for each of the three different cell lines. (**B**) Screenshot of *MPRIP* as an example of a gene that shows an overrepresentation of the proximal APA isoform in the cytoplasmic fraction, relative to the nuclear fraction, in all three colorectal cell lines analysed here. (**C**) Shows the number of significant UTR-APA shortening and lengthening events occurring when comparing nuclear fractions from each of the three different cell lines. (**D**) Screenshot of *TOLLIP* as an example of a gene that shows shortening in the nucleus when comparing 1CT to SW480 or SW620 cells without affecting the cytoplasmic APA profile highlighting the importance of using fractionation to make any predictions on the potential physiological impact of the profile changes observed. B&D), Read numbers in reads per million (RPM) and cell lines are indicated to the left of each track. C1, C2: cytoplasmic fractions repeat 1 and 2. N1 and N2: nuclear fractions repeat 1 and 2. Tracks show reads from sequencing the 3′ends of extracted RNAs. Gene structure, gene names and the position of the proximal (pPA) and distal (dPA) poly(A) sites are indicated above the tracks and blue arrow indicates the orientation of the respective gene. Screenshots were generated using IGV, with ‘window function’ set to ‘Mean’ and Auto scale’.

**Figure 2.**
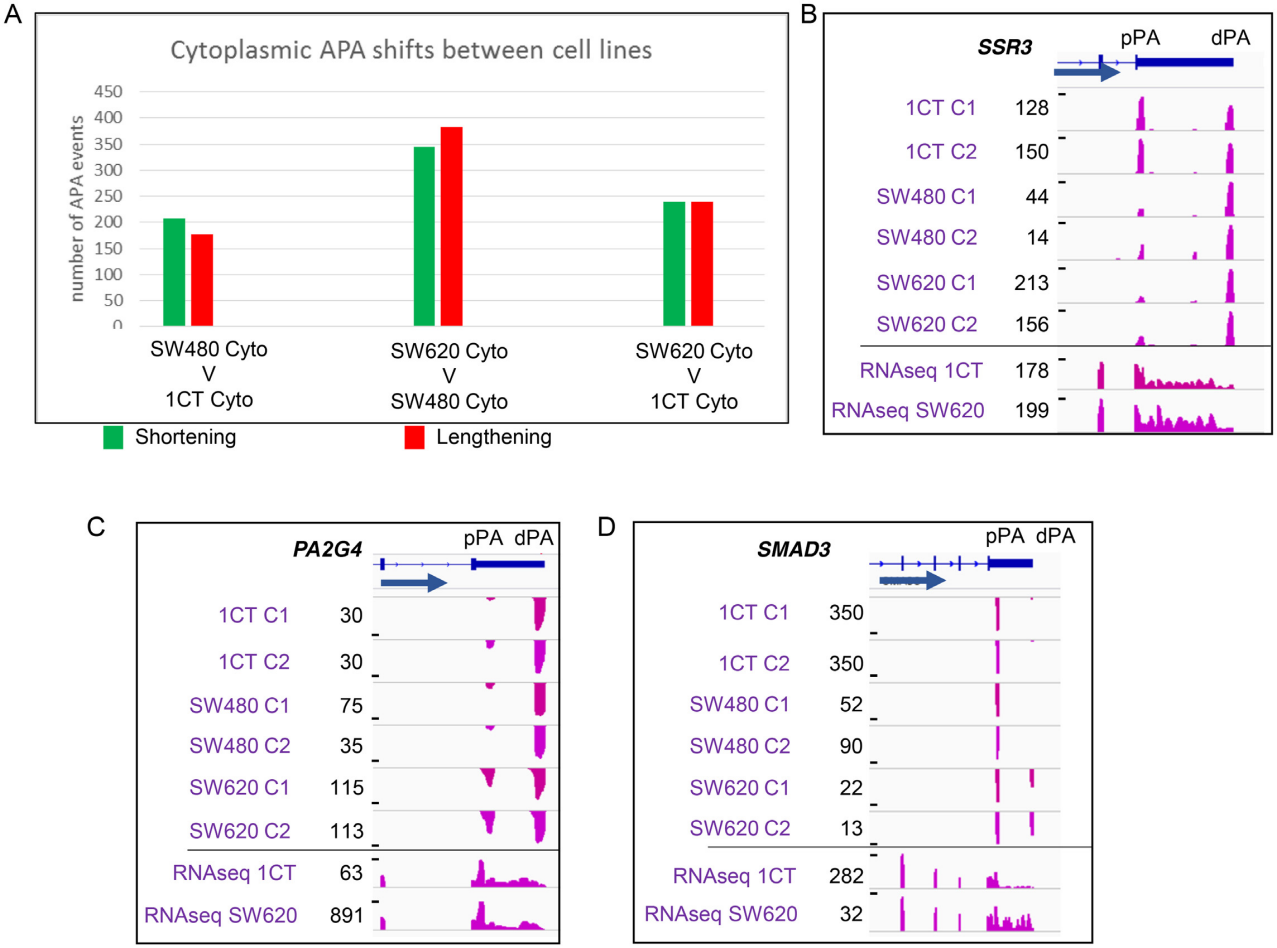
Comparison of UTR-APA abundances of the cytoplasmic fractions between the different cell lines. (**A**) The identity of the samples being compared is shown under each comparison with each bar representing events where the shorter APA isoforms (green) or longer APA isoforms (red) have a significantly higher relative representation in the first sample. Significant differentially represented APA isoforms are those in which *P*≤ 0.01 (Fisher's exact test) and the isoform abundance change is ≥5% relative to total APA isoforms associated with that gene. No clear trend towards either shortening (green) or lengthening (red) is apparent when the cytoplasmic APA profiles between the three different cell lines are compared. Panels B–D: Example genome browser pictures showing cell line specific changes in the overrepresentations of a particular UTR-APA isoform. Read numbers and respective cell lines are indicated to the left of each track. C1, C2: cytoplasmic fractions repeat 1 and 2. Tracks show reads from sequencing the 3′ends of extracted RNAs. Gene structure, gene names and the position of the proximal (pPA) and distal (dPA) poly(A) sites are indicated above the tracks and blue arrow indicates the orientation of the respective gene. Bottom two tracks represent full RNA-seq of SW620 and 1CT cytoplasmic polyadenylated mRNA. (**B**) *SSR3* as an example gene that undergoes lengthening in both SW480 and SW620 when compared to 1CT cells. (**C**) *PA2G4* and (**D**) *SMAD3* as examples that undergo shortening (*PA2G4*) or lengthening (*SMAD3*) in SW620 cells compared to both SW480 and 1CT cells.

We first compared the UTR-APA profiles of the subcellular fractions for each cell line. For this, the biological replicates of each sample were combined and analysed for significant differential representation of APA isoforms. We classified significant APA events as those where *P* ≤ 0.01 (Fisher's exact test). Initially, comparisons between the cytoplasmic and nuclear UTR-APA profiles were compared for each of the 1CT, SW480 and SW620 cell lines (Figure 1A, Supplementary Figure S5). For UTR-APA, this showed a very similar distribution to that seen in the HEK293 and HBL cells (13) with the cytoplasmic fractions generally showing an overrepresentation of the proximal APA isoforms in the cytoplasm versus the nucleus as exemplified by *MPRIP* (Figure 1B). The nuclear and cytoplasmic comparisons within the cells confirmed the robustness of the fractionation approach and we thus proceeded to compare the nuclear and the cytoplasmic fractions between the different cells with the aim to isolate UTR-APA events that may contribute to cancer progression. To isolate cell-specific changes in poly(A) site selection, we next compared the nuclear APA profiles of either SW480 or SW620 to 1CT cells. This analysis showed a slight bias towards a shift to the proximal poly(A) sites in both SW620 and SW480 when compared to 1CT cells. (Figure 1C, Supplementary Figure S6A). However, as the example of *TOLLIP* demonstrates (Figure 1D), many APA events that occur in the nucleus do not necessarily transmit into the cytoplasm and so may have limited physiological impact. In order to capture potentially more relevant APA events between the cell lines we next focused on the cytoplasmic fractions. Whilst we found hundreds of APA profile changes for each comparison, no global biases to either shortening (overrepresentation of proximal APA isoforms) or lengthening (overrepresentation of distal APA isoforms) were found (Figure 2A, Supplementary Figure S6B). To get a sense of how early during carcinogenesis particular APA profiles may be established, we counted the events that change between SW480 and 1CT cells and also occur in the comparison between SW620 and 1CT cells. Of the 240 statistically significant genes that undergo lengthening between SW620 and 1CT, 9% also lengthen between SW480 and 1CT, as exemplified by *SSR3* (Figure 2B). Conversely, of the 239 shortening events between SW620 and 1CT, 13% also shorten between SW480 and 1CT. These events thus may represent APA profile changes that occur early during the transition from normal to adenocarcinoma cells and persist in the SW620 metastatic cells. Interestingly, this cohort of genes include *OTUB1* (26) (lengthening) and *ATP2A2* (27) (shortening) (Supplementary Figure S7A) that have both been associated with colon cancer. We then isolated APA profile changes that are unique events occurring only in the metastatic derived SW620 cells and identified 124 lengthening and 117 shortening events. Most interestingly, in both of these cohorts we extracted genes that are linked to proliferation, including *PA2G4* and *SMAD3* (Figure 2C and D, Supplementary Figure S8A and B). *PA2G4 (EBP1)* is involved in the regulation of cell growth and has been shown to be upregulated in colorectal cancers where it may counteract the tumour suppressor E2F1 (28). *SMAD3* is part of the TGF-β signalling pathway and disruption of the *SMAD3* gene in mouse results in the onset of metastatic colorectal cancer (29). The above described approach enabled us to isolate a significant number of nuclear and cytoplasmic APA events that are characteristic for each cell line, including events at genes that have been linked to colon cancer.

### *MTHFD1L* and *NAP1L1* show characteristic coding region APA profile changes in metastasis-derived cells

**Figure 3.**
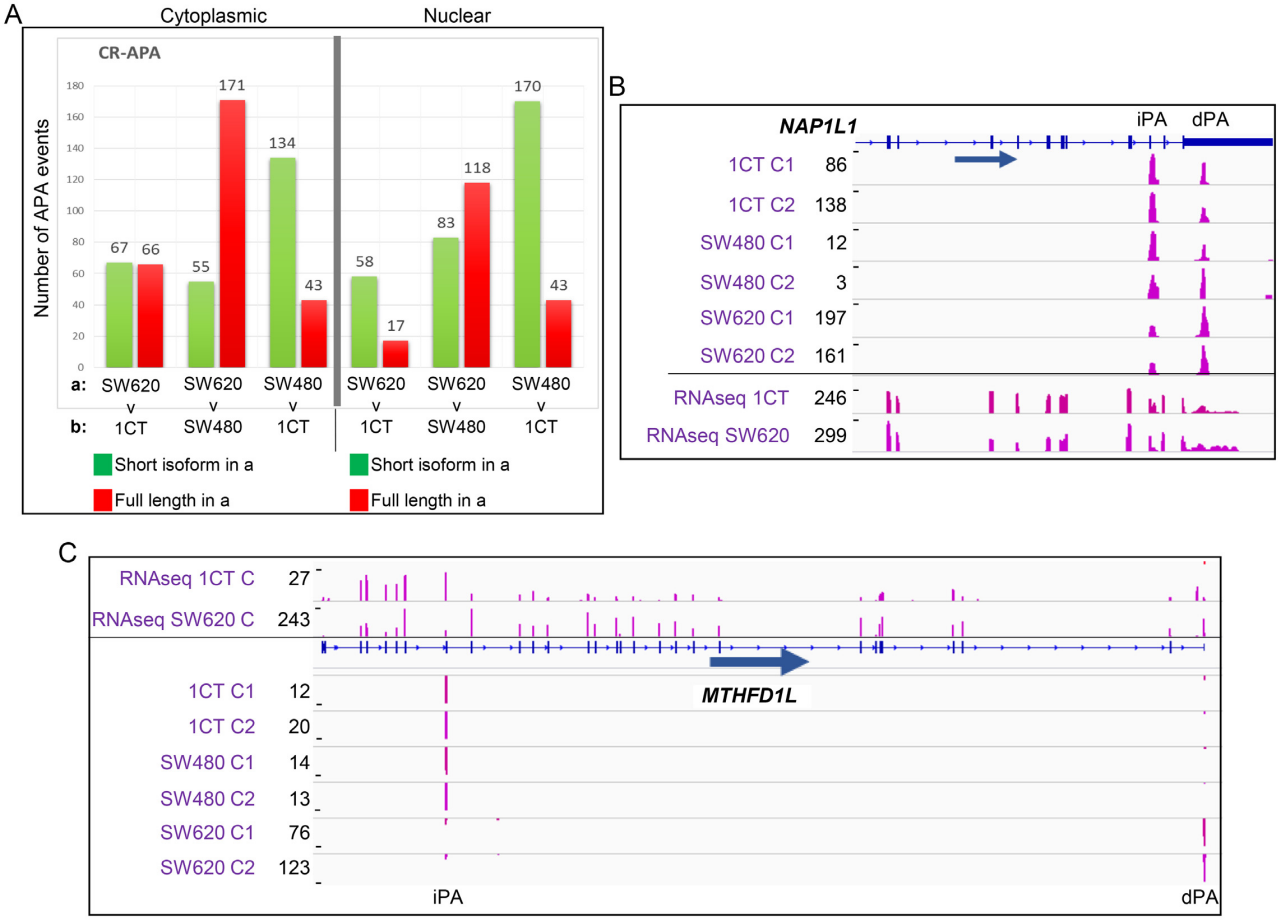
Comparison of CR-APA events across the three cell lines. (**A**) CR-APA profile changes when the cytoplasmic fractions (panel on the left) and nuclear fractions (panel on the right) are compared between the different cell lines. The identity of the samples being compared is shown under each comparison with each bar representing events where the shorter isoforms generated using internal poly(A) sites (green) or longer APA isoforms generated using distal poly(A) sites resulting in transcripts encoding the full-length protein (red) have a significantly higher relative representation in the first sample. Significant differentially represented APA isoforms are those in which *P*≤ 0.01 (Fisher's exact test) and the isoform abundance change is ≥5% relative to total APA isoforms associated with that gene. (**B**) *NAP1L1* as an example that shows an SW620 specific overrepresentation of the full-length isoform in the cytoplasmic fraction. Top six tracks show reads from sequencing the 3′ ends of extracted cytoplasmic RNAs. Bottom two tracks show reads from full RNA-seq of SW620 and 1CT cytoplasmic polyadenylated mRNA. Gene structure and an arrow indicating the orientation of the gene are shown below the tracks. C1, C2: cytoplasmic fractions repeat 1 and 2. dPA: distal poly(A) site. IPA: internal poly(A) site. (**C**) *MTHFD1L* undergoes SW620 specific CR-APA with higher frequency of shortened transcripts in 1CT cells compared to SW620 cells where the full-length isoform is dominant. Bottom six tracks show reads from sequencing the 3′ ends of extracted cytoplasmic RNAs. Top two tracks show reads from full RNA-seq of SW620 and 1CT cytoplasmic polyadenylated mRNA. Gene structure and an arrow indicating the orientation of the gene are shown. C1, C2: cytoplasmic fractions repeat 1 and 2. dPA: distal poly(A) site. IPA: internal poly(A) site.

To identify additional potentially physiologically relevant APA events, we next interrogated CR-APA profile changes that occur between the three cell lines. Compared to UTR-APA, CR-APA events are more likely to have a physiological impact as the resulting mRNA isoforms have differential coding capacities. Unlike for UTR-APA, characteristic global profile movements between SW480 and 1CT and SW620 and 1CT cells were evident when the cytoplasmic (Figure 3A, left panel, Supplementary Figure S9A) and nuclear fractions (Figure 3A, right panel; Supplementary Figure S9B) were compared. In the nucleus, there appears to be a strong tendency towards usage of the internal poly(A) sites in SW620 and SW480 cells compared to the normal colon epithelial 1CT cells. Conversely, when the nuclear fractions of SW620 and SW480 cells are compared there appears to be a tendency to over represent the full-length APA isoform in the metastatic compared to the adenocarcinoma derived cells. With the exception of the comparison between SW620 and 1CT the cytoplasmic profile trends are similar to nuclear ones (Figure 3A, left panel). Compared to the UTR-APA analysis, overall fewer CR-APA profile changes are identified when the fractions of the different cell lines are compared but importantly, this cohort includes genes that have been strongly linked to cancer progression; most notably *MTHFD1L* and *NAP1L1*. *NAP1L1* is a nucleosome assembly protein that is overexpressed in pancreatic neuroendocrine neoplasm metastases (30) and *MTHFD1L* encodes a key component of the mitochondrial tetrahydrofolate cycle (mTHF) which has been implicated in proliferation (31). In metastatic derived cells both *NAP1L1* (Figure 3B) and *MTHFD1L* (Figure 3C, Supplementary Figure S8C), show an overrepresentation of the full-length mRNA versus the shorter isoform and this for *MTHFD1L*, although notoriously difficult to establish between cell lines, appears to be matched at the protein level (Supplementary Figure S10A and B). There is also remarkably little overlap between the CR-APA events that occur between SW620 and 1CT and SW480 and 1CT cells (Supplementary Figure S11), indicating that in each cell line a specific set of genes are undergoing CR-APA. From this analysis, we conclude that we have identified a number of cell type specific CR-APA events and this includes genes, most notably *MTHFD1L*, that have a high relevance for cancer progression.

### Enrichment of hnRNPC binding sites in aUTRs of genes that use distal poly(A) sites in metastasis derived cells

**Figure 4.**
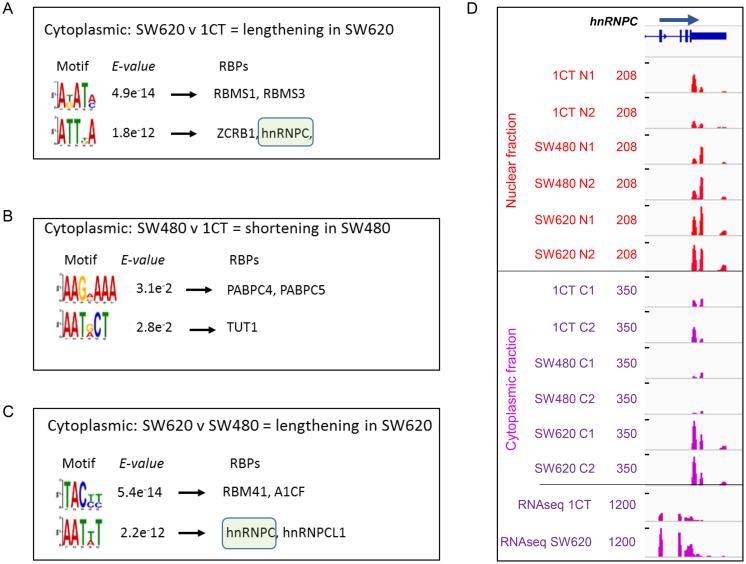
Motif analysis and interacting RBPs identified in aUTRs extracted from APA isoforms that show differential APA profiles when cytoplasmic APA isoforms are compared between: (**A**) isoforms that lengthen in SW620 compared to 1CT and (**B**) that shorten in SW480 compared to 1CT and (**C**) lengthen in SW620 compared to SW480. In all cases the top 2 significant overrepresented motifs were interrogated using the TOMTOM motif comparison tool to identify the corresponding interacting human RNA binding proteins with the default significance threshold set at *E*-value <10. The top two RBP hits for each motif are shown. No motifs were found for comparisons of SW620 compared to 1CT cells shortening in former and SW620 compared to SW480 cells shortening in former and SW480 compared to 1CT cells lengthening in former. Green boxes highlight hnRNPC hits. (**D**) *hnRNPC* is overexpressed in the nuclear (red) and cytoplasmic (purple) fractions of SW620 compared to SW480 and 1CT cells at the RNA level. Top 12 tracks show reads from sequencing the 3′ ends of extracted nuclear RNAs (tracks 1–6) or cytoplasmic RNAs (tracks 7–12). Bottom two tracks show reads from full RNA-seq of SW620 and 1CT cytoplasmic polyadenylated mRNA. C1, C2: cytoplasmic fractions repeat 1 and 2. N1, N2: nuclear fractions repeat 1 and 2. Read numbers are scaled, tracks 1–6 max = 208 RPM, tracks 7–12 max = 350 RPM, tracks 13–14 max = 1200 RPM.

After isolation of the above described CR-APA and UTR-APA events we explored which regulatory pathways are responsible for shaping these specific profiles. To that end, we examined the cytoplasmic-specific alternative UTRs (aUTRs) of genes that show distinct shifts in more detail. The aUTRs of genes that showed differential UTR-APA profiles in the cytoplasm between SW620 and 1CT cells and SW620 cells and SW480 cells were extracted and the aUTRs that changed in the same direction in the nuclear fraction were subtracted to bias the search towards factors that cause APA events in the cytoplasm. The DREME motif analysis pipeline (19) was used to identify significantly enriched (*E* value < 0.05) motifs in the aUTR sequences of the genes that either lengthen or shorten their UTRs in each pairwise comparison between cell types. The aUTRs of genes that move in the opposite direction served as control sequences. Extracted motifs (Figure 4A–C) were subsequently interrogated using the TOMTOM motif comparison tool to identify the corresponding interacting human RNA binding proteins (Figure 4A–C, RBPs column) as specified by the RNAcompete method (21). No significant motifs were identified in aUTRs from genes that have an overrepresentation of the shorter UTR-APA isoforms in SW620 versus both 1CT and SW480 cells. In contrast, the aUTRs extracted from genes that have an overrepresentation of the longer UTR-APA isoform in SW620 versus 1CT and SW480 cells both revealed enriched motifs that qualify as target sites for a variety of RNA binding proteins (RBPs) (Figure 4A–C). Most interestingly, a sequence that is significantly enriched in both aUTR lengthening cohorts when SW620 cells are compared to either 1CT or SW480 cells qualifies as a binding site for hnRNPC. hnRNPC has been associated with the regulation of m6A regulated splicing (32), stabilizing RNAs (33,34), suppressing exonization of Alu elements (35) and most recently has also been implicated in APA regulation (36). hnRNPC thus represents a potential APA regulator that may be involved in shaping APA profiles during cancer progression. However, if hnRNPC does contribute to the observed lengthening in SW620 cells, it could be expected that the expression of hnRNPC is ‘de-regulated’ in SW620 compared to SW480 and 1CT cells. To address this possibility, we compared the gene expression levels of *hnRNPC* between the different cell lines. *hnRNPC* is significantly overexpressed (Fisher's exact test, *P*≤0.01) in SW620 cells compared to both SW480 and 1CT cells (Figure 4D, Supplementary figure S10A (bottom panel) and E) and thus supports the hypothesis that it may regulate APA of a subset of genes during cancer progression.

### Knock down of hnRNPC reverts a subset of SW620 APA profiles to the 1CT profiles

**Figure 5.**
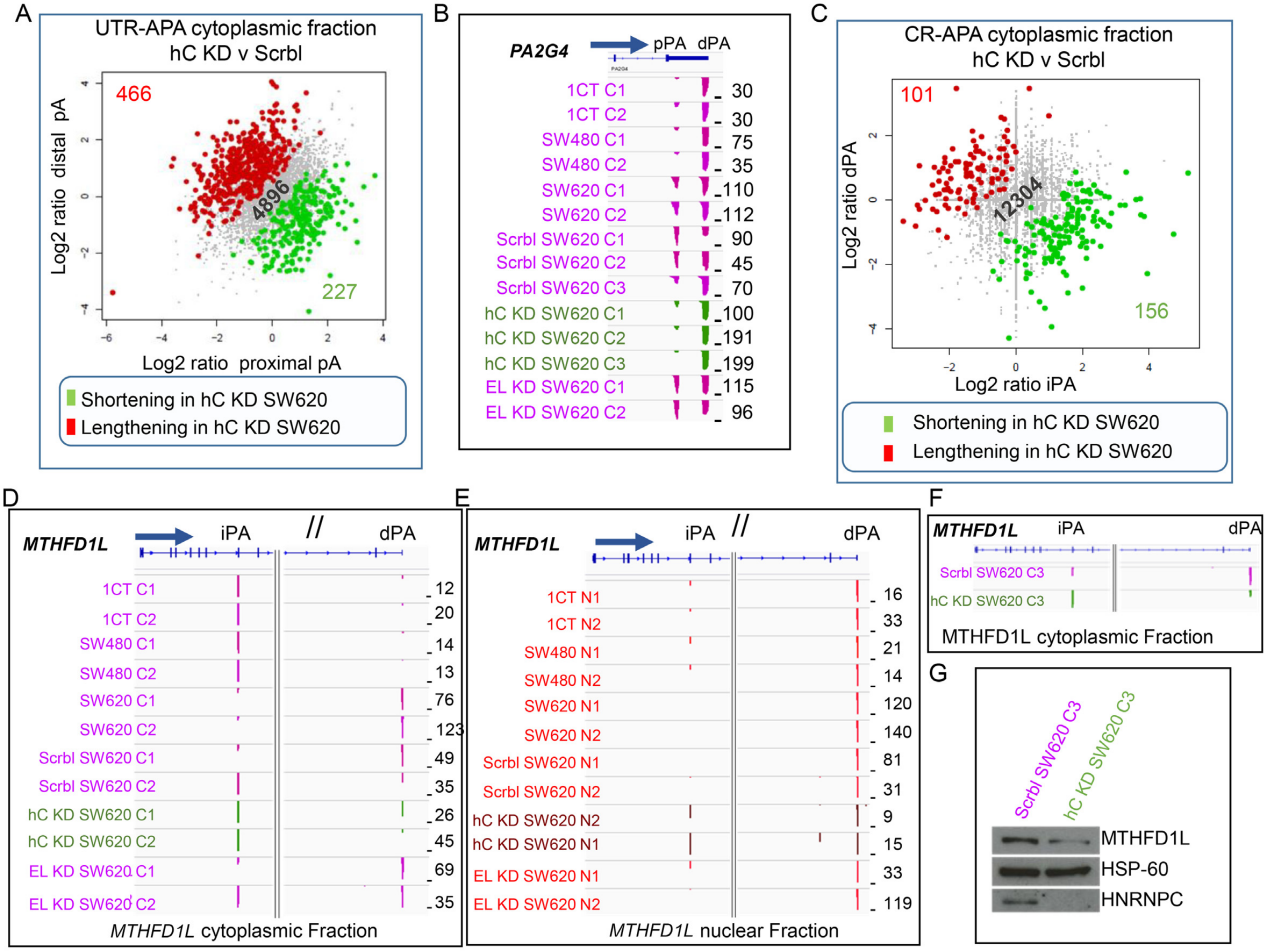
hnRNPC knock down reverts SW620 specific UTR-APA and CR-APA changes to profiles characteristic for SW480 and 1CT cells. (**A**) Scatter plot of UTR-APA events in the cytoplasm of SW620 cells transfected with siRNAs targeting hnRNPC compared to SW620 cells transfected with scrambled siRNAs (combined replicates). Genes are highlighted when the shorter (green) or longer (red) isoform have a significantly higher representation (Fisher's exact *P*≤ 0.01) in SW620 cells with reduced levels of hnRNPC compared to SW620 cells with normal hnRNPC levels. Grey dots represent genes with UTR-APA isoforms that do not significantly differ in frequency between the nucleus and the cytoplasm. (**B**) *PA2G4* representing an example of a gene that reverts the cytoplasmic APA profiles upon hnRNPC depletion in SW620 cells to profiles characteristic for SW480 and 1CT cells. Scrbl SW620 C1 and C2 SW620 cells transfected with scrambled siRNAs and hC KD SW620 C1 and C2 green tracks represent SW620 cells transfected with siRNAs targeting hnRNPC mRNA, EL KD SW620 C1 and C2 represent SW620 cells transfected with siRNAs targeting *ELAVL1* mRNA. (**C**) Scatter plot of CR-APA events in the cytoplasm of SW620 cells transfected with siRNAs targeting hnRNPC compared to SW620 cells transfected with scrambled siRNAs (combined replicates). Genes are highlighted when the shorter (green) or longer (red) isoform have a significantly higher representation (Fisher's exact *P*≤ 0.01) in SW620 cells with reduced levels of hnRNPC compared to SW620 cells with normal hnRNPC levels. (A, C) Grey dots represent genes with APA isoforms that do not significantly differ in frequency between the nucleus and the cytoplasm. The actual numbers of genes undergoing relative shortening (green) or lengthening (red) or genes where isoforms do not change in frequency (grey) are indicated in the bottom right and top left corners and in the centre respectively. (**D**) Cytoplasmic CR-APA profile changes in *MTHFD1L* as a result of hnRNPC depletion (green tracks) in SW620 cells and (**E**) changes in the nuclear fraction (brown tracks). For both (D) and (E), the left panel shows the 5′ region of the *MTHFD1L* gene and right panels show the 3′ region of the gene. (**F**) Genome browser shot focusing on the cytoplasmic APA changes of the third independent biological repeat for CR-APA in the *MTHFD1L* gene. (**G**) Western blot of total protein extracts targeting full-length *MTHFD1L* (top panel) and hnRNPC (bottom panel) in SW620 treated with scrambled siRNAs or siRNAs targeting hnRNPC. Middle panel shows the HSP-60 loading control.

To verify a potential contributory role of hnRNPC in shaping a SW620 characteristic APA profile, we reduced hnRNPC levels in SW620 cells by siRNA mediated knock down (kd). If elevated hnRNPC levels in SW620 cells drive specific APA profiles it can be expected that some of the APA profiles revert to those more characteristic of 1CT and SW480 cells. An additional control was included that consisted of the kd of the related *ELAVL1* mRNA. Significant kd was achieved for both proteins (Supplementary figure S12). As previously shown for HEK293 cells (36), kd of hnRNPC (3 combined repeats) results in a general increase in UTR length in both the nuclear and cytoplasmic fractions when compared to SW620 cells transfected with a scrambled siRNA (Figure 5A, Supplementary Figure S13A). Of the 127 genes that lengthen in both the SW620 v 1CT and the SW620 v SW480 cytoplasmic cohorts, seven genes revert and shorten upon hnRNPC kd, as exemplified by *CLCN7* (Supplementary Figures S14A, S15). Genes that undergo shortening in both the SW620 v 1CT and SW620 v SW480 comparisons were also affected. Of the 119 shortening genes, 15 genes revert to the 1CT/SW480 profile and showed lengthening of their UTRs upon hnRNPC kd. Most interestingly, this cohort also included *PA2G4* (Figure 5B, Supplementary Figure S14B). From this analysis we conclude that the elevated levels of hnRNPC in SW620 cells may contribute to the establishment of characteristic UTR-APA profile changes for a subset of genes.

To assess whether hnRNPC levels also affect CR-APA profiles we next focused on CR-APA events in hnRNPC kd SW620 cells. As can be seen in Figure 5C and Supplementary Figure S13B under reduced levels of hnRNPC, intragenic poly(A) sites are favoured in both the nuclear and to a lesser extent in the cytoplasmic fractions. In the cytoplasmic fraction, we identified 101 genes that undergo statistically significant lengthening (relative increase of frequencies of the full-length isoform compared to the short isoform generated by usage of the intragenic poly(A) site), when hnRNPC levels are reduced in SW620 cells (Figure 5C, Supplementary Figure S16A). We also extracted 156 genes that have the opposite trend showing a statistically significant increase in the frequency of the short isoforms upon hnRNPC kd (Figure 5C, Supplementary Figure S16B). Interestingly, GO analysis of the 27 genes that lengthen in SW620 cells compared to both 1CT and SW480 (Supplementary Figure S16C) using the Enrichr platform (37,38), identifies an enrichment for folate associated metabolic pathway genes (Supplementary Figure S16D). Strikingly, upon hnRNPC depletion, four genes (Supplementary Figure S16B, text box on right), including *MTHFD1L* (Figure 5D, F) ‘revert’ to the 1CT and SW480 characteristic profiles. A similar trend, albeit not statistically significant, is also apparent in the nucleus (Figure 5E). These changes appear to be specific to hnRNPC as no change in the profiles of these genes is seen, when ELAVL1 is knocked down instead (Figure 5B and C and D & E). Importantly, when hnRNPC is knocked down in SW620 cells we not only observe a change in *MTHFD1L* APA profiles (Figure 5F), but this is concomitant with a reduction in MTHFD1L protein level (Figure 5G, Supplementary Figure S19). From this analysis, we conclude that elevated levels of hnRNPC in SW620 cells affect the MTHFD1L protein levels by modulating CR-APA.

### *MTHFD1L, PA2G4, CLCN7* and *SMAD3* APA profile changes are also seen in tumour derived RNA-seq samples

**Figure 6.**
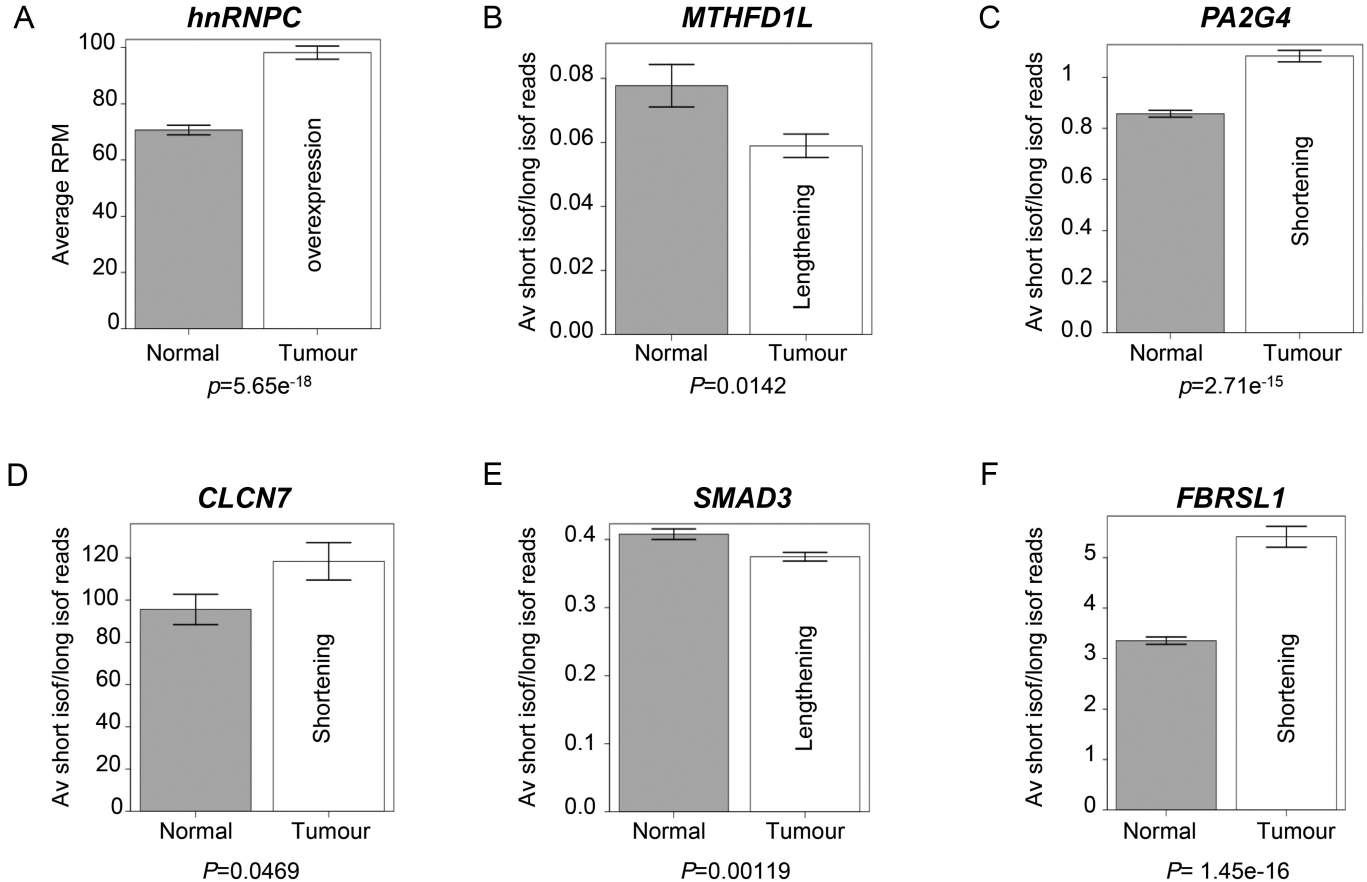
Comparison of APA profile shifts in RNAseq samples extracted from matched patient normal and tumour samples (34). (**A**) Bar chart comparing the mean read numbers aligning to the terminal exon of *hnRNPC* per million total aligned reads in normal and tumour samples. (**B**) Bar chart comparing the mean ratios of reads aligning to the terminal exon of the short and long isoforms of *MTHFD1L* in normal and tumour samples. (C–F) Bar charts comparing the mean ratios of reads aligning to the terminal exon of the short isoform and the aUTR for genes (**C**) *PA2G4*, (**D**) *CLCN7*, (**E**) *SMAD3* and (**F**) *FBRSL1* for normal and tumour samples. All bar chart error bars show the S.E.M. *P*-values (Student's *t*-test) test the significance of the difference in the means.

Our approach using distinct cell lines identified characteristic APA profile changes in several cancer relevant genes including *SMAD3, PA2G4* and *MTHFD1L* and linked the latter two to the de-regulation of hnRNPC in metastatic site-derived cells. Whilst the cell line-based approach is ideal to elucidate potentially relevant APA profile changes and underlying mechanisms, we wanted to verify whether these changes also occur in actual tumours. To that end we used a recently published RNA-seq data set generated from tumour and normal colon epithelial cell samples derived from 103 patients (22). Using these data, we determined the mean reads per million overlapping with the *hnRNPC* 3′ exon across all tumour samples and across all normal samples (Supplementary Figure S18). Similar to the cell-based system, this analysis revealed elevated expression of *hnRNPC* in the tumour compared to the normal samples (Figure 6A). We then used the same approach to calculate the ratios of reads mapped to the terminal exon of the short isoform and the terminal exon of the full-length *MTHFD1L* isoform for each sample and then compared the mean of the ratios across all normal samples to that for all tumour samples. Again, similar to the observations we made between SW620 and 1CT cells, this analysis suggests that the short isoform is overrepresented in the normal versus the tumour-derived samples. We also calculated the ratios of read numbers between short and long 3′ UTR isoforms of, *PA2G4* (Figure 6C), *CLCN7* (Figure 6D) *SMAD3* (Figure 6E), *FBRSL1* (Figure 6F), *OTUB1* (Supplementary Figure S6B) and *ATP2A2* (Supplementary Figure S6C) which matches the trends observed when APA of these genes was compared in the three cell lines (Figure 2 and 3 and Supplementary Figure S7A and D). These data underpin the relevance of the characteristic APA profile changes and their underlying mechanisms that we identified using the tissue culture-based cell system.

## DISCUSSION

We have used a subcellular fractionation approach to monitor APA profile changes during carcinogenesis using a cell line-based model system that mimics colon cancer progression. Our aim was not just to log APA events in the different cell lines but rather isolate APA events that are likely to have a physiological impact and elucidate the underlying molecular mechanisms. Assessing UTR-APA profile changes in the cytoplasm allowed us to identify differential expression of hnRNPC as a critical cell-type-specific regulator of UTR- and CR-APA events. We found that elevated levels of hnRNPC in metastatic-derived colon cancer cells are responsible for driving CR- and UTR-APA of a subset of genes including *MTHFD1L*, which has been strongly linked to cancer progression. This clearly highlights the relevance of our initial finding made in the cell line-based colon cancer model. We show that a reduction of hnRNPC levels in metastatic-derived SW620 cells reverts the APA profile of several genes including *MTHFD1L* to those characteristic for normal- and adenocarcinoma-derived cells. Importantly, both the increased expression of *hnRNPC* and the concomitant APA profile changes were confirmed when RNA-seq data of patient-derived tumour and non-tumour colon samples were compared. We thus not only present evidence that APA can make a critical contribution to colon cancer progression but also provide an underlying molecular mechanism by identifying deregulation of hnRNPC expression as a critical novel regulator of cancer specific APA for a subset of genes.

A link between proliferation and APA has long been recognised (39) and APA profile changes in the context of cancer have been extensively studied in the past decade (40,7,11,10). Whilst significant prognostic power has been attributed to the changing APA profiles observed in tumour samples (8,10) the physiological impact that APA has, however, is still largely uncertain. Furthermore, the underlying molecular mechanisms by which cancer-specific APA profiles are established is poorly understood and only a few cancer-specific regulators such as CFIm25 (7) and E2F (41) have so far been identified. We have not only found that hnRNPC can be deregulated in tumours driving characteristic APA profile changes but we further show that some of the affected genes are of high physiological importance for cancer progression. In particular, the hnRNPC mediated regulation of *MTHFD1L* illustrates the significance of our finding as it is a critical enzyme of mitochondrial folate metabolism which is tightly linked to cell proliferation. The overexpression of both *MTHFD1L* and *MTHFD2* has been reported in cancer cells where it is associated with greater mortality. Elevated expression of these mitochondrial enzymes is believed to play a significant role in accelerating the synthesis of one-carbon units required for nucleotide synthesis and synthesis of glycine, thereby abetting cell proliferation (31). Our results suggest that hnRNPC could contribute to the establishment of an elevated *MTHFD1L* level by regulating APA of this gene. As a strictly nuclear hnRNP, elevated hnRNPC levels are likely to affect *MTHFD1L* APA at the point of poly(A) site selection by competing for binding with CstF64 at the DSE of the internal poly(A) site selection. This scenario is plausible as e-Clip data (ENCODE; GEO:GSE91860) shows hnRNPC binding at the 3′UTR and in the DSE region of the internal poly(A) site but not at the distal poly(A) site (Supplementary Figure S17). The physiological consequence of this APA event may contribute to the acceleration of the tetrahydrofolate pathway in the metastatic cells. The shift from the short isoform to the full-length may be critical as the short isoform of *MTHFD1L* has been shown to produce a non-functional version of MTHFD1L (42). Therefore, elevated levels of hnRNPC in tumours, through the above described mechanisms, may contribute to the increased levels of *MTHFD1L* which in turn creates a metabolic environment that is beneficial for proliferation.

As shown in Figure 5B and Supplementary Figure S15, the elevated levels of hnRNPC also affect UTR-APA by influencing the poly(A) site selection of *PA2G4* and *CLCN7*. Interestingly, both *MTHFD1L* and *CLCN7* show similar profile changes in Hek293 cells upon hnRNPC depletion (Supplementary Figure S20). However, for all APA events, the true physiological contribution of the characteristic profile changes can be difficult to determine and this is particularly the case for UTR-APA events. We cannot exclude the possibility that many of the hnRNPC induced UTR-APA changes are ‘collateral damage’ and may have little, if any, effect on the cellular state and actual disease progression.

The fact that *SMAD3* undergoes APA, may be of significance in the context of colon cancer progression. SMAD3 protein is a transforming growth factor-β (TGF-β) receptor, and disruption of the *SMAD3* gene in mouse results in the onset of metastatic colorectal cancer after 4–6 months (29). In normal epithelial tissue, TGF-β signalling provides a growth inhibitory signal, so downregulation or disruption of receptors on this pathway reduce or remove these inhibitory signals, thus increasing the rate of proliferation in these cells (43). The distal mRNA isoform may be less efficiently translated and so contribute to overall suppression of *SMAD3* expression. The increased frequency of the longer *SMAD3* APA isoform in the cytoplasm of SW620 cells compared to both 1CT and SW480 cells may be regulated by MiR-145. MiR-145 has previously been shown to target the *SMAD3* 3′UTR (44). Interestingly, in SW480 cells, which show the same *SMAD3* APA profile as 1CT cells (Figure 2C), MiR-145 is downregulated 19-fold compared to SW620 cells (45), providing a possible explanation for the relative increase of the longer isoform compared to the shorter isoform in the cytoplasm of SW620 cells.

## DATA AVAILABILITY

Data accession number: GSE102357. URL: http://www.ncbi.nlm.nih.gov/geo/query/acc.cgi?acc=GSE102357.

## Supplementary Material

gkz461_Supplemental_Files
