# Supplementary material for: hnRNPC regulates cancer-specific alternative cleavage and polyadenylation profiles
Source: Nucleic Acids Res. 2019 May 31;47(14):7580–91. doi: 10.1093/nar/gkz461 (PMC6698646; doi:10.1093/nar/gkz461)
Supplement: gkz461_Supplemental_Files [file gkz461_supplemental_files.docx]

Supplementary Data


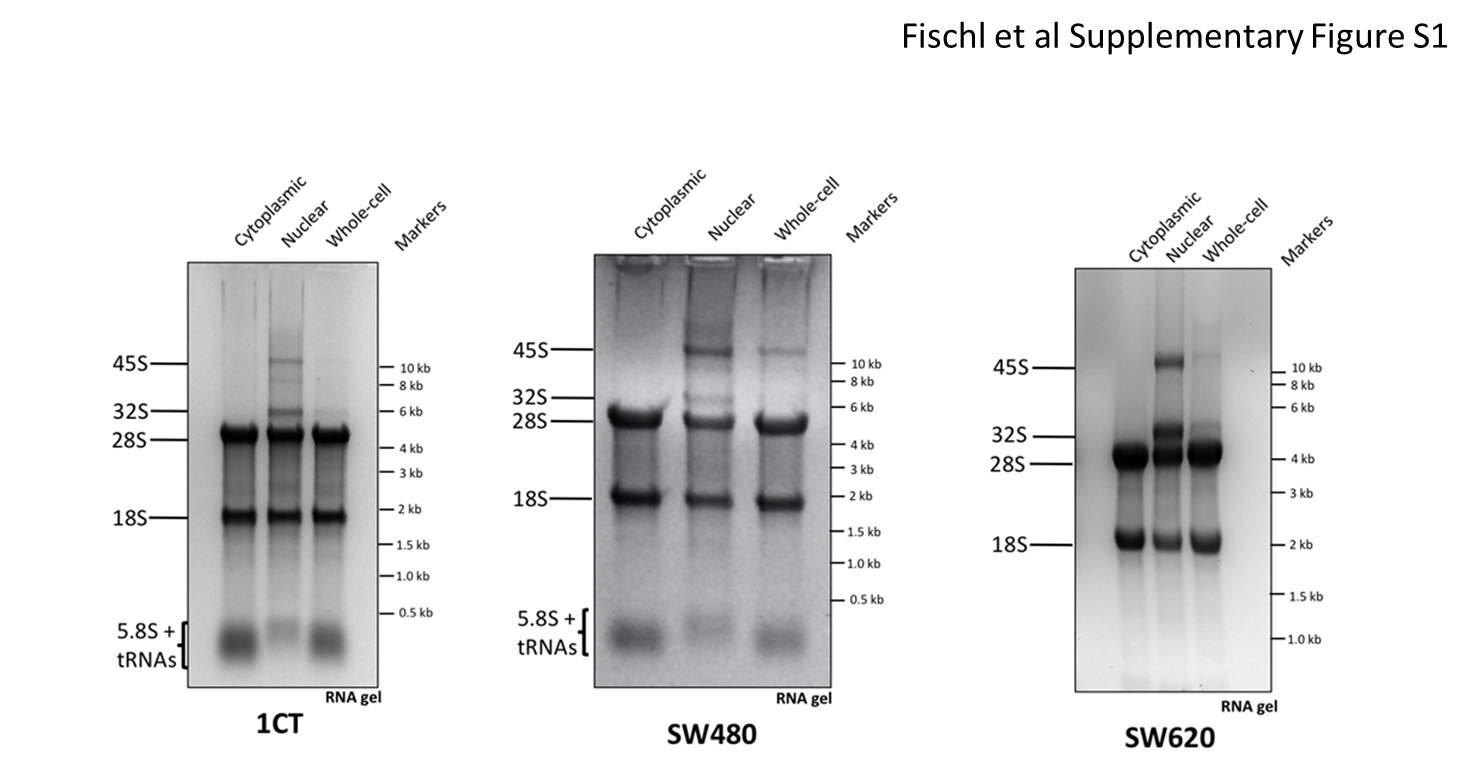


**Supplementary Figure S1**: **Quality control assessment of subcellular RNA fractions**. Cytoplasmic, nuclear and whole cell RNA isolated from 1CT, SW480 and SW620 cells were assessed by gel electrophoresis and scored for the presence of rRNA precursors in the respective fractions. DNA size markers are indicated to the right of the gels and the rRNA species are shown on the left of each gel. The 32S and 45S rRNA precursors are only visible in the nuclear and faintly in whole cell but are absent in cytoplasmic fractions indicating successful fractionation.


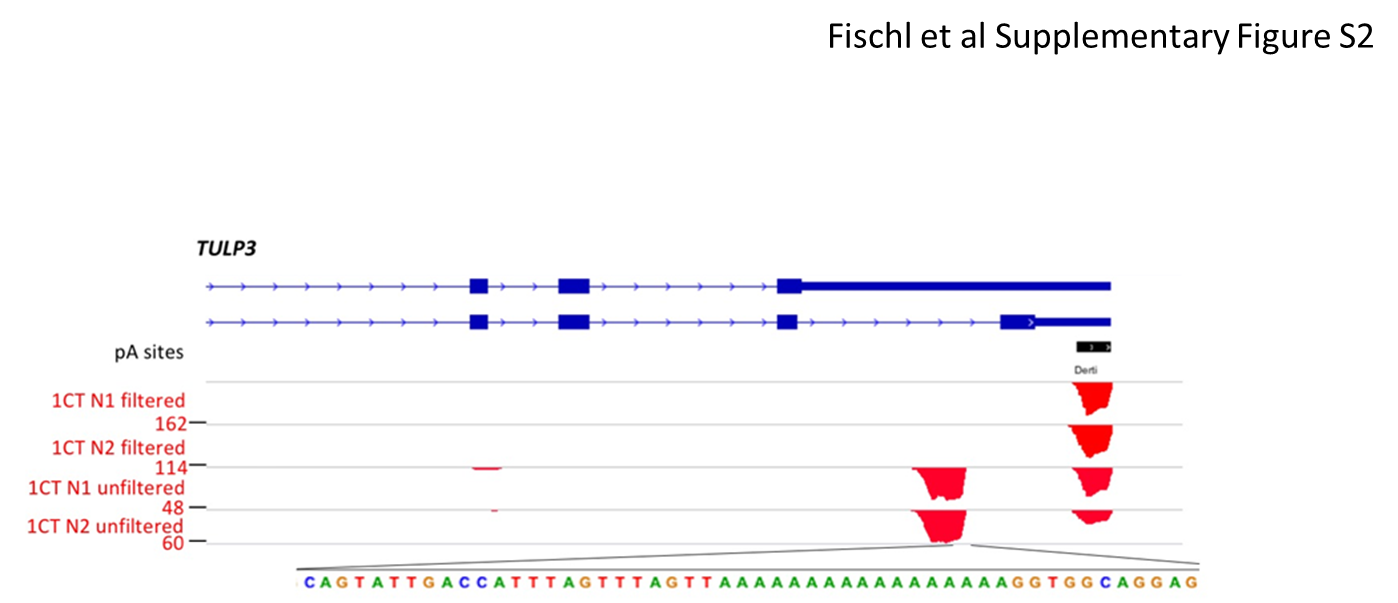


**Supplementary Figure S2**: **Isolation of reads mapping to true pA sites.** A comparison of Bigwig files at the *TULP3* locus generated from BAM files before and after being filtered for internal priming events. Filtering was performed using the Bedtools Intersect function which removed all reads that did not overlap with a region 100 nt directly upstream of a previously identified poly(A) site. These regions are shown in the track directly under the schematic of the *TULP3* gene. The sequence region at the end of the additional peak in the unfiltered sample has been expanded and shows a long stretch of genomically encoded adenosines indicating that these reads are likely produced as a result of internal priming. As these reads do not overlap with any previously identified poly(A) site, these reads are filtered out and not used in the APA analysis.


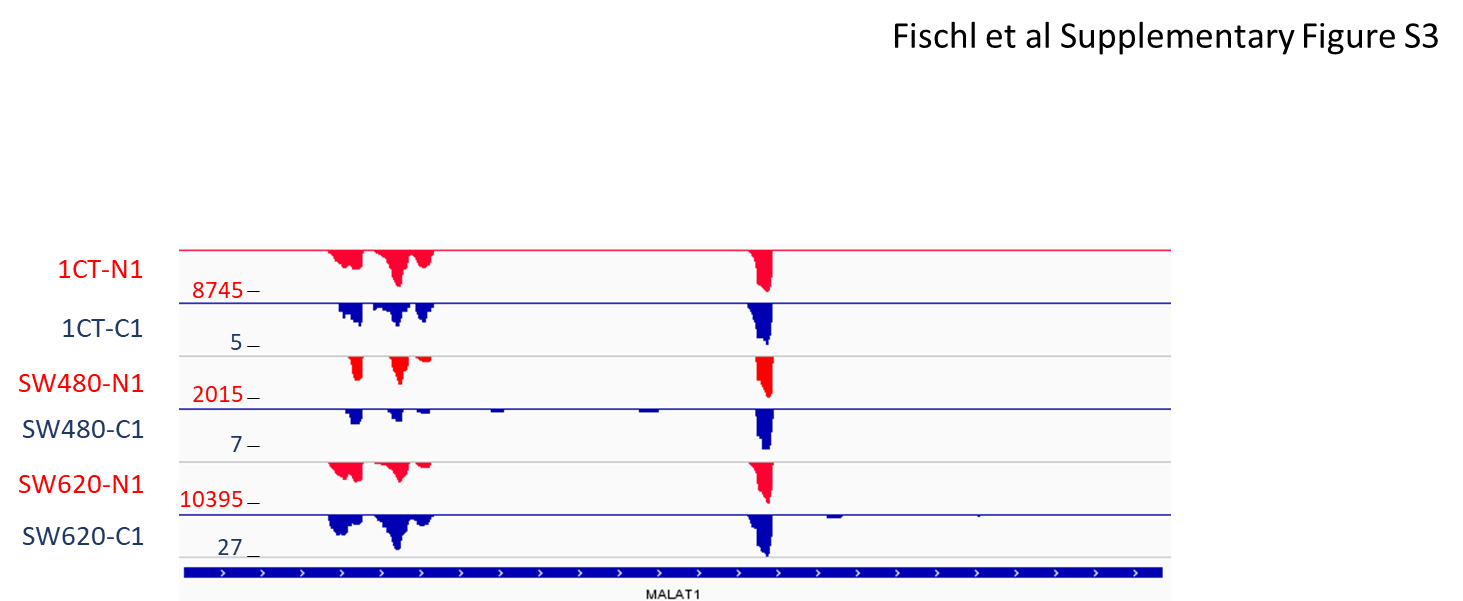


**Supplementary Figure S3**: **Quality control of subcellular fraction at the RNAseq level.** The strictly nuclear non-coding RNA MALAT1 serves as a measure for fractionation purity. Tracks for the first repeat of nuclear (N1, red) and cytoplasmic (C1, blue) Quantseq 3’ RNA-seq reads that map to MALAT1 are shown for each of the three cell lines. The read numbers in reads per million are shown to the left confirming that the cytoplasmic fraction is free of nuclear contaminants.


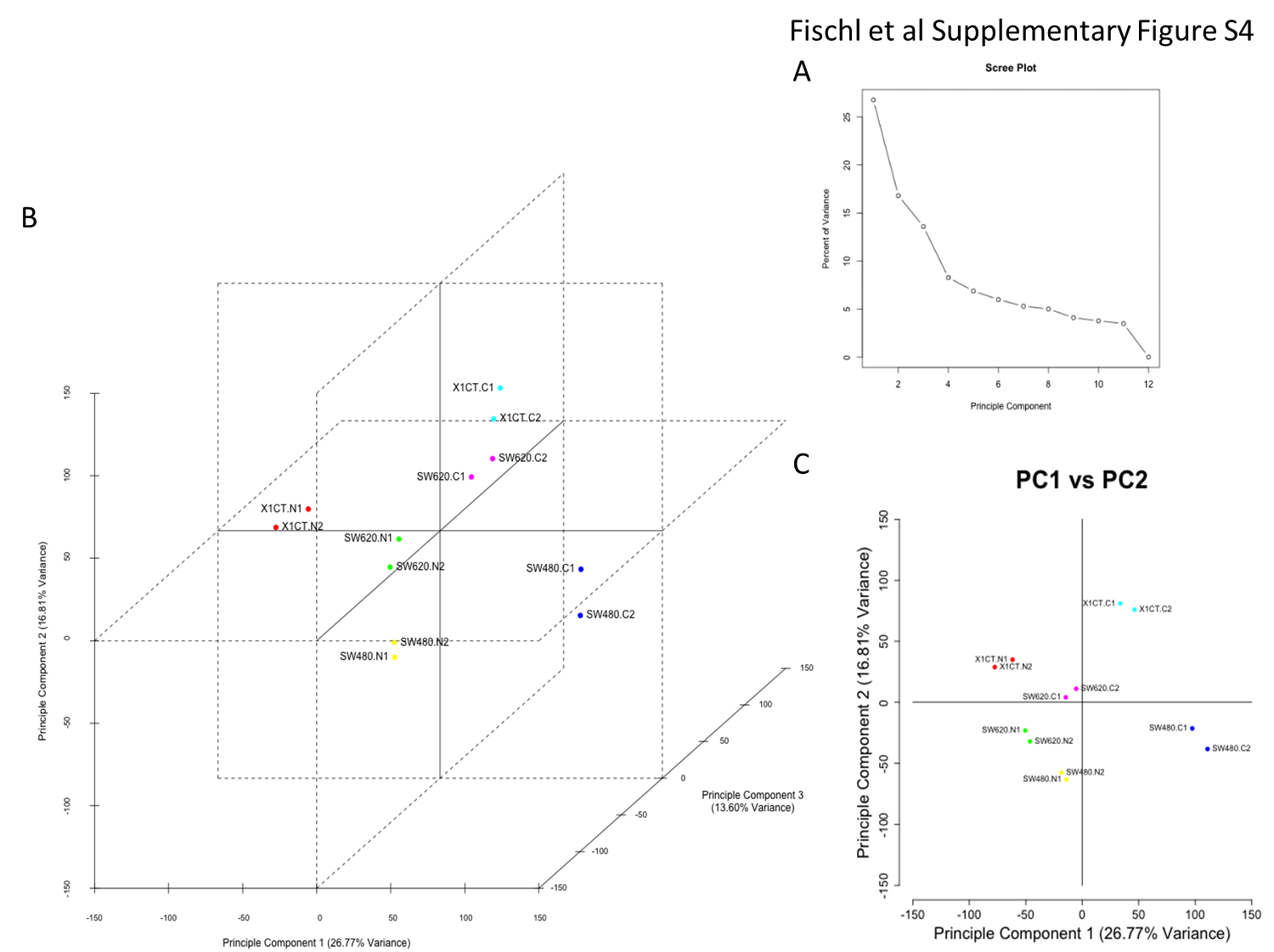


**Supplementary Figure S4, Principal component analysis of sequencing replicates.**

PCA of 1CT (X1CT), SW480 and SW620 subcellular fractions using log2 transformed gene expression values of two independent biological repeat experiments. **A**) Scree plot depicting % variances of the 12 components, **B**) PCA plot using component 1 and 2 and 3 **C**) PCA plot of component 1 and 2, as per scree plot in A.


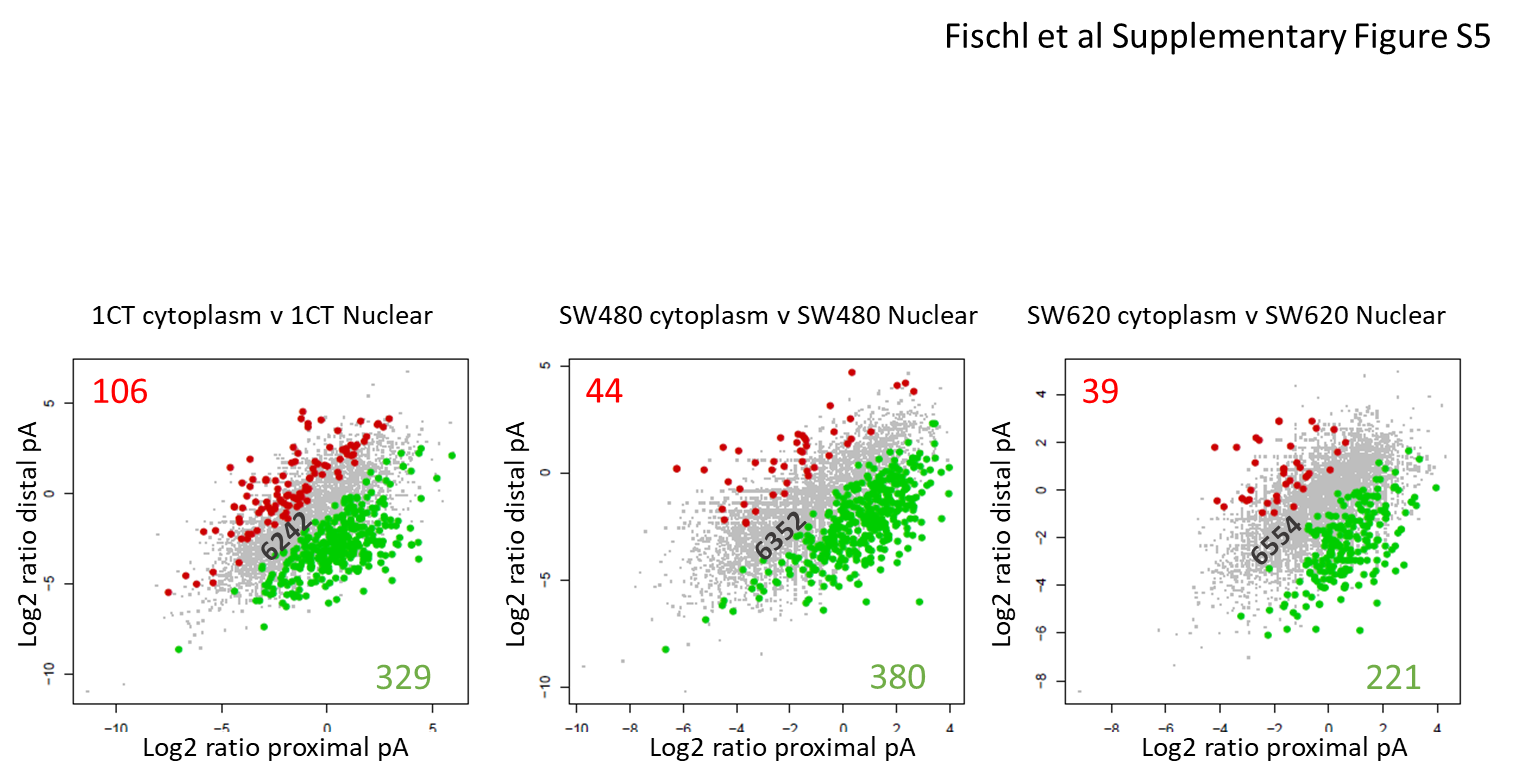


**Supplementary Figure S5. Scatter plot of UTR-APA events in the nucleus and cytoplasm for 1CT, SW480 and SW620 cells** (combined replicates)**.**

Genes with UTR-APA isoforms that differ significantly in relative representation of their most highly expressed APA isoforms in the cytoplasm compared to the nucleus in 1 CT cells (left panel) SW480 cells (middle panel) and SW620 cells (right panel). Genes are highlighted when the shorter (green) or longer (red) isoform have a significantly higher representation (Fisher exact p≤0.01 in the cytoplasm compared to the nucleus. Grey dots represent genes with UTR-APA isoforms that do not significantly differ in frequency between the nucleus and the cytoplasm.


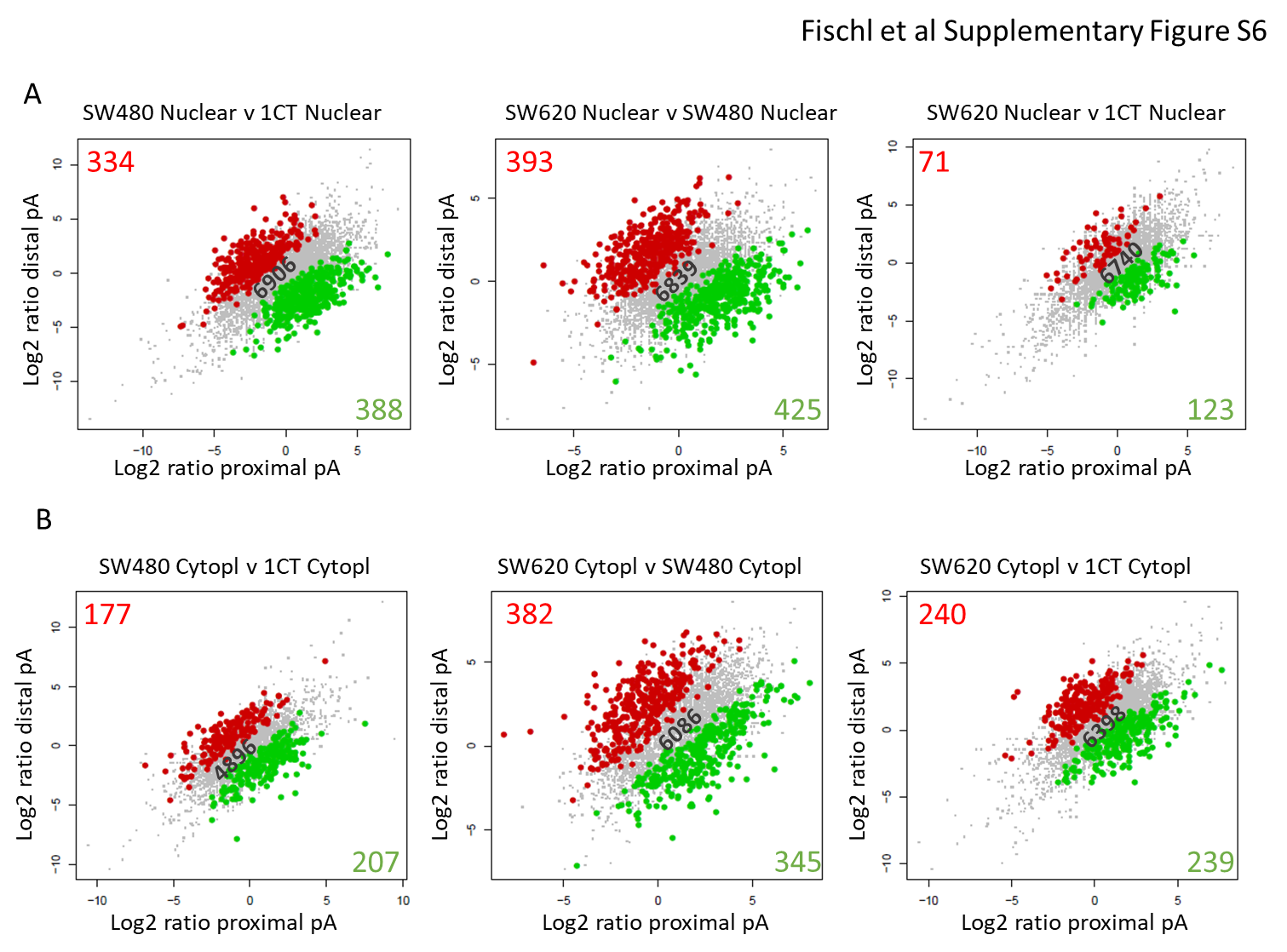


**Supplementary Figure S6. Scatter plots comparing UTR-APA of nuclear and cytoplasmic fractions between the different cell lines** (combined replicates)**.**

**A)** Genes with UTR-APA isoforms that differ significantly in relative representation of their most highly expressed APA isoforms in the nucleus of SW480 cells compared to nuclear fraction in 1CT cells (left panel), nuclear faction of SW620 cells compared to nuclear fraction of SW480 cells (middle panel) and nuclear fraction of SW620 cells compared to the nuclear fraction of 1CT cells (right panel). **B)** as in A) but comparing UTR-APA changes of the cytoplasmic fractions between the three cell lines. For **A** and **B**, genes are highlighted when the shorter (green) or longer (red) isoform have a significantly higher representation (Fisher exact p≤0.01) in the nuclear fraction of SW620 compared to 1CT, SW620 compared to SW480 and SW480 compared to 1CT cells respectively. Grey dots represent genes with UTR-APA isoforms that do not significantly differ in frequency in the corresponding fractions between the cell lines. The number of genes undergoing relative shortening (green) or lengthening (red) or genes where isoforms do not change in frequency (grey) are indicated in the bottom right and top left corners and in the centre respectively.


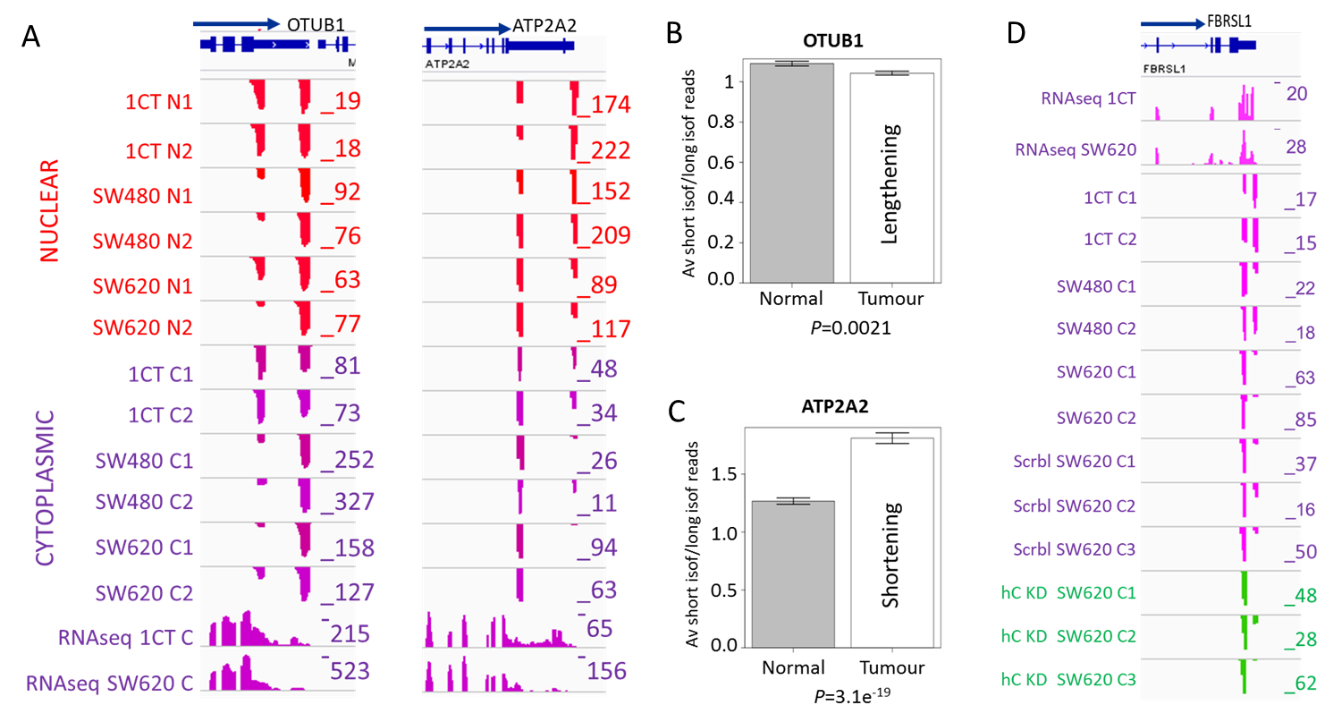


**Supplementary Figure S7**: **Cell type specific APA profiles in OTUB1, ATP2A2 and FBRSL1.**

**A**) Nuclear and cytoplasmic tracks of 3’ extracted reads and full RNA-Seq reads mapped to OTUB1 (left) and ATP2A2 (right) showing a lengthening APA event in the former and a shortening event in the latter in SW620 and SW480 cells compared to 1CT cells. To the left of the tracks the identification of each cell line and subcellular localisation (C1, C2: cytoplasmic repeat1 and 2; N1, N2: nuclear repeat 1 and 2) are shown and the corresponding read numbers for each track are indicated on the right. **B** and **C**) Evaluation of APA using RNAseq data (Ongen et al. 2014) compiled from normal and colon tumour samples isolated from patients. Mean ratios of read numbers mapped to the 3’exon (short isoform) or the aUTR of the mRNA (long isoform) in normal and tumour derived RNA-seq samples (Ongen et al., 2014) confirms a similar lengthening event at OTUB1 (B) and shortening event at ATP2A2 (C) as observed in SW620 cells compared to 1CT cells. All bar chart error bars show the S.E.M. p-values (Student’s t-test) test the significance of the difference in the means. **D)** FBSRL1 as an example that shows cytoplasmic RNA shortening in SW620 cells versus 1CT cells which is independent of hnRNPC levels. The shortening in SW620 cells cannot be “reversed” when hnRNPC levels are reduced by siRNA KD. Scrbl SW620C1/C2/C3 = three cytoplamsmic replicates of SW620 cells treated with “scrambled” control siRNAs. hC KD SW620C1/C2/C3 = three cytoplasmic replicates of SW620 cells treated with siRNAs targeting hnRNPC.


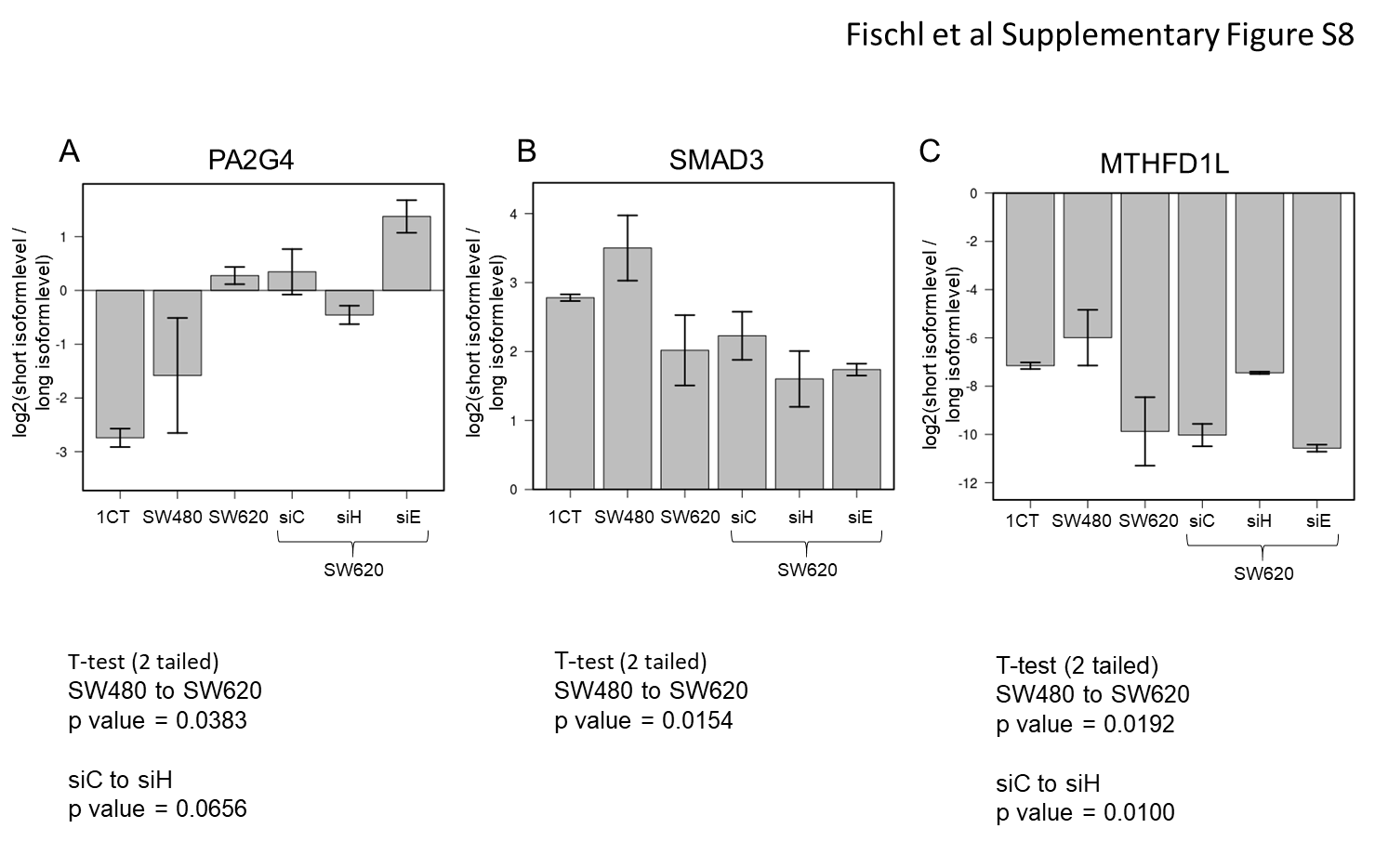


**Supplementary Figure S8. Validation of MTHFD1L, SMAD3 and PA2G4 alternative poly(A) site usage by qRT-PCR. A&B&C)** RNA from the cytoplasmic fractions of 1CT (CT) SW480, SW620, SW620 subjected to scrambled control siRNA (siC) or siRNAs targeting hnRNPC (siH) or ELAVL1 (siE) respectively was reversed transcribed and the frequencies of the short and long APA isoforms were assessed by qPCR. Bar charts show the mean log2 ratio of short to long transcript isoforms averaged across all biological replicates for *PA2G4* (**A**), *SMAD3* (**B**) and *MTHFD1L* (**C**). Error bars show the standard deviation of the independent replicates (n=2 1CT and siE / n≥3 for all other conditions). Student t-test p values for the respective comparisons are indicated below the graphs.


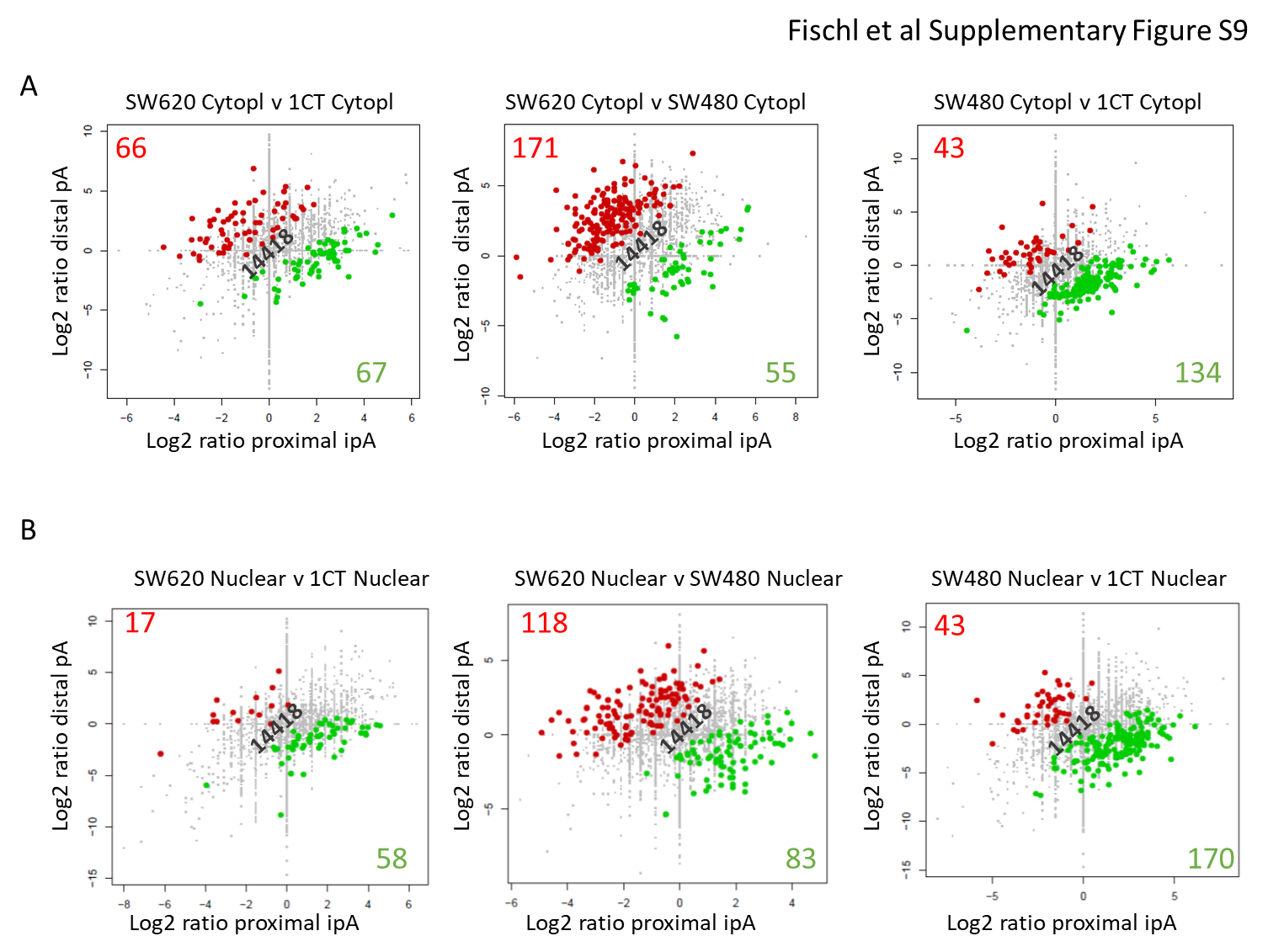


**Supplementary Figure S9. Scatter plots comparing CR-APA of nuclear and cytoplasmic fractions between the different cell lines** (combined replicates)**.** **A)** Genes with CR-APA isoforms that differ significantly in relative representation of their proximal (intronic and exonic) APA isoforms relative to the 3’most exon (distal) isoforms in the nuclear fraction of SW480 cells compared to the nuclear fraction in 1CT cells (left panel), the nuclear faction of SW620 cells compared to the nuclear fraction of SW480 cells (middle panel) and the nuclear fraction of SW620 cells compared to the nuclear fraction of 1CT cells (right panel). **B)** as in A but comparing CR-APA changes of the cytoplasmic fractions between the three cell lines. For **A** and **B**, Genes are highlighted when the shorter proximal (green) or full-length distal (red) isoform have a significantly higher representation (Fisher exact p≤0.01) in SW620 compared to 1CT, in SW620 compared to SW480 and SW480 compared to 1CT cells). Grey dots represent genes with CR-APA isoforms that do not significantly differ in frequency. The number of genes undergoing relative shortening (green) or lengthening (red) or genes where isoforms do not change in frequency (grey) are indicated in the bottom right and top left corners and in the centre respectively.


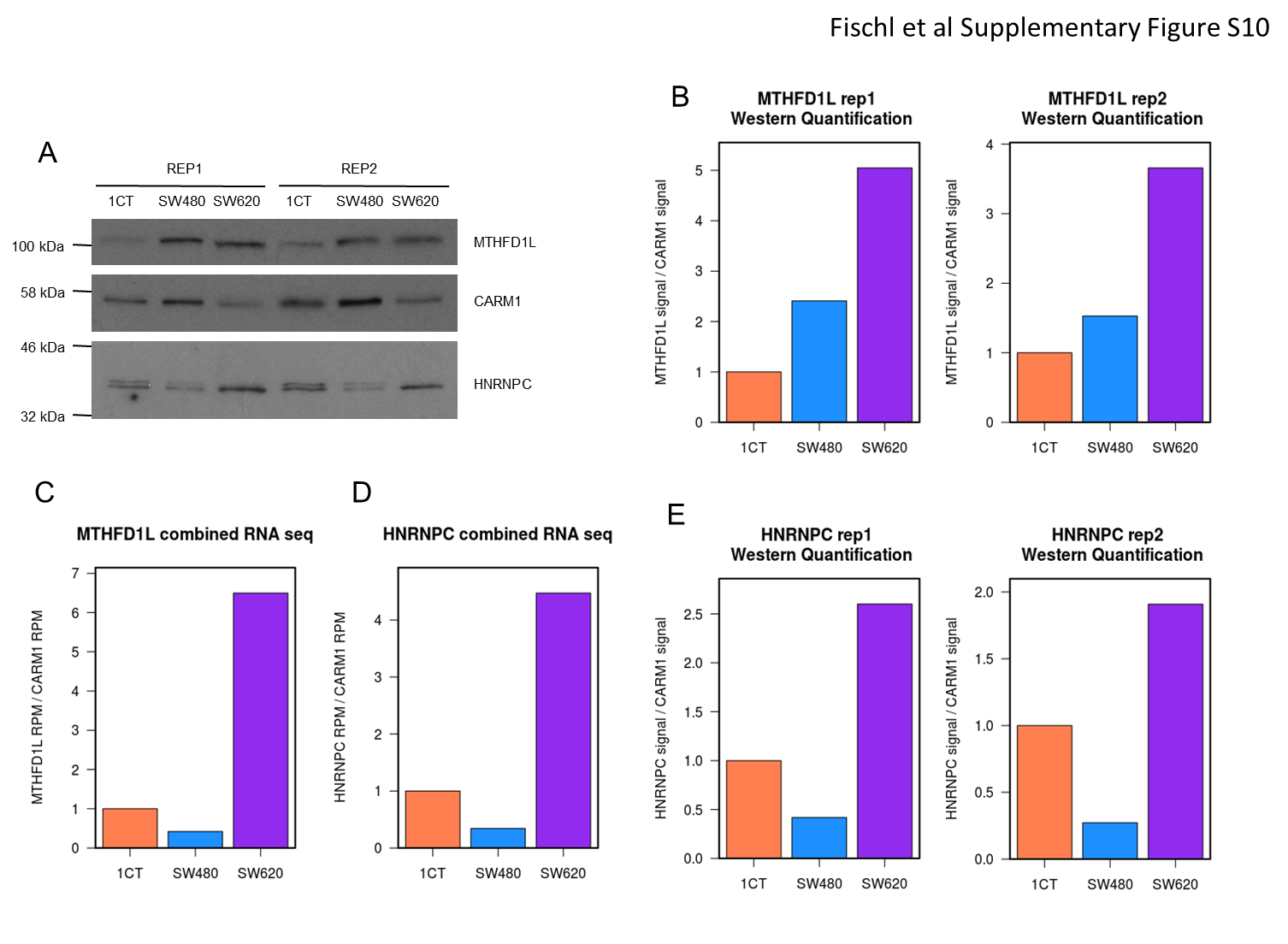


**Supplementary Figure S10. Attempt to compare of MTHFD1L and hnRNPC protein levels by western blot across cell lines using RNAseq levels as a reference. A)** Western blot using antibodies targeting MTHFD1L (top panel), CARM1 (middle panel) and hnRNPC bottom panel. The MW standards are indicated to the left of the panels and two replicates for each cell line as indicated above the panels are shown. **B)** quantitation (signal of MTHFD1L / CARM1) of the western blots for replicates 1 (rep1) and 2 (rep2) are plotted for each cell line. **C)** RNAseq ratio of reads per million of MTHFD1L / CARM1 from the combined replicates. **D)** RNAseq ratio of reads per million of hnRNPC / CARM1 from the combined replicates. **E)** quantitation (signal of hnRNPC / CARM1) of the western blots for replicates 1 (rep1) and 2 (rep2) are plotted for each cell line.


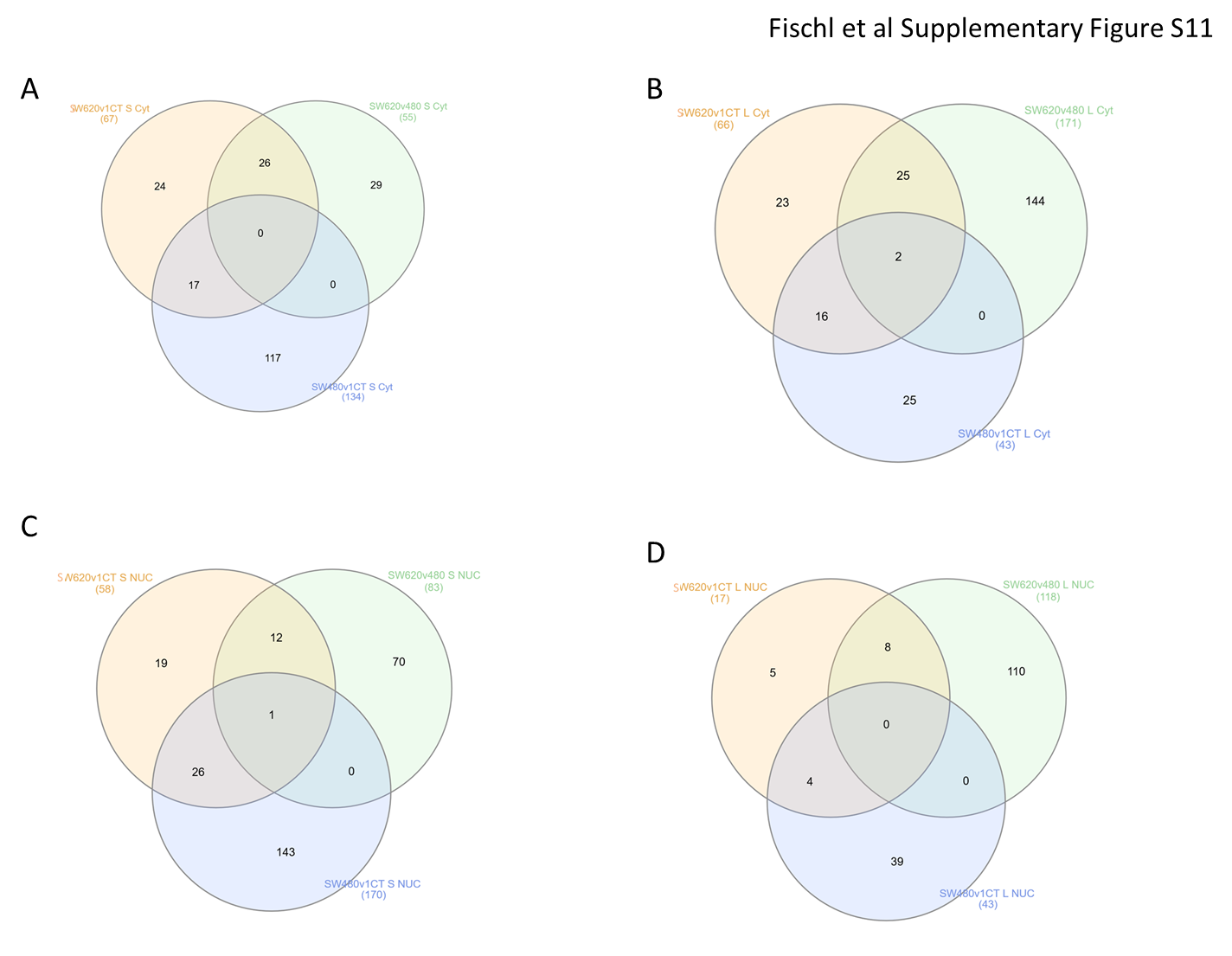


**Supplementary Figure S11**: Minimal overlap between the CR-APA events between the different cell lines. **A)** Venn diagram showing the overlaps between the CR-APA SHORTENING (S) (increase of frequencies of internal poly(A) APA isoforms compared to distal isoforms) events in the cytoplasm between the SW620 v 1CT, SW620 v SW480 and SW480 v 1CT cohorts. **B)** Venn diagram showing the overlaps between the CR-APA LENGTHENING (L) (increase of frequencies of distal poly(A) APA isoforms compared to proximal isoforms) events in the cytoplasm between the SW620 v 1CT, SW620 v SW480 and SW480 v 1CT cohorts. **C)** Venn diagram showing the overlaps between the CR-APA SHORTENING (S) (increase of frequencies of internal poly(A) APA isoforms compared to distal isoforms) events in the nucleus between the SW620 v 1CT, SW620 v SW480 and SW480 v 1CT cohorts. **D)** Venn diagram showing the overlaps between the CR-APA LENGTHENING (L) (increase of frequencies of distal poly(A) APA isoforms compared to proximal isoforms) events in the nucleus between the SW620 v 1CT, SW620 v SW480 and SW480 v 1CT cohorts.


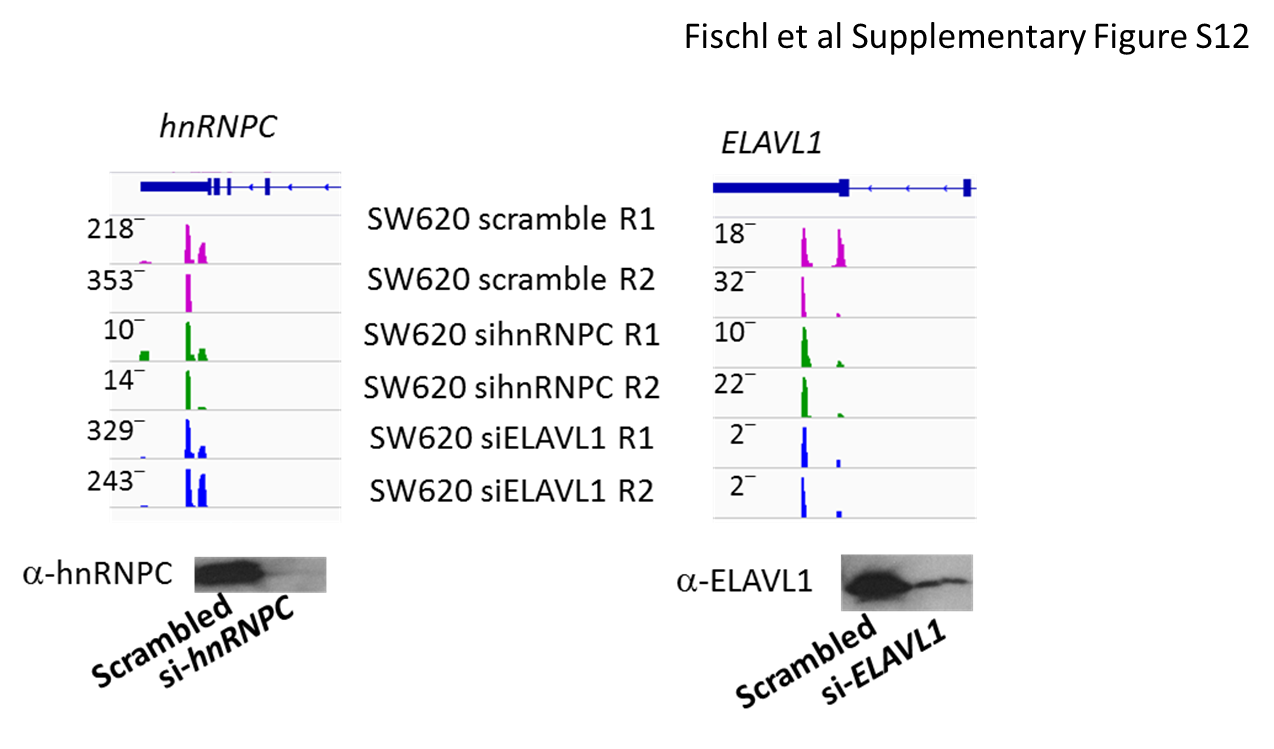


**Supplementary Figure S12**: **siRNA mediated knock down (KD) of hnRNPC and ELAVL1 in SW620 cells. Top panels:** Two replicates (R1 and R2) of SW620 cells were transfected with a scrambled siRNA (scramble) or siRNAs that are targeting either hnRNPC (sihnRNPC) or ELAVL1 (siELAVL1) respectively. Cells were subsequently fractionated and 3’extracted RNA was sequenced for APA analysis. The successful KD of hnRNPC can be seen at the RNAseq level. Compared to SW620 cells that were transfected with control scrambled siRNAs (SW620 scramble, purple tracks) reads mapped to hnRNPC or ELAVL1 collapse in cells that were treated with sihnRNPC (green tracks) or siELAVL1 (blue tracks). The tracks show cytoplasmic RNA. **Lower panels**: The reduction in hnRNPC and ELAVL1 at the mRNA level is matched at the protein level as verified by western blot using antibodies specific to hnRNPC (left hand side) and ELAVL1 (right hand side) confirming a significant reduction of the two proteins after siRNA mediated KD.


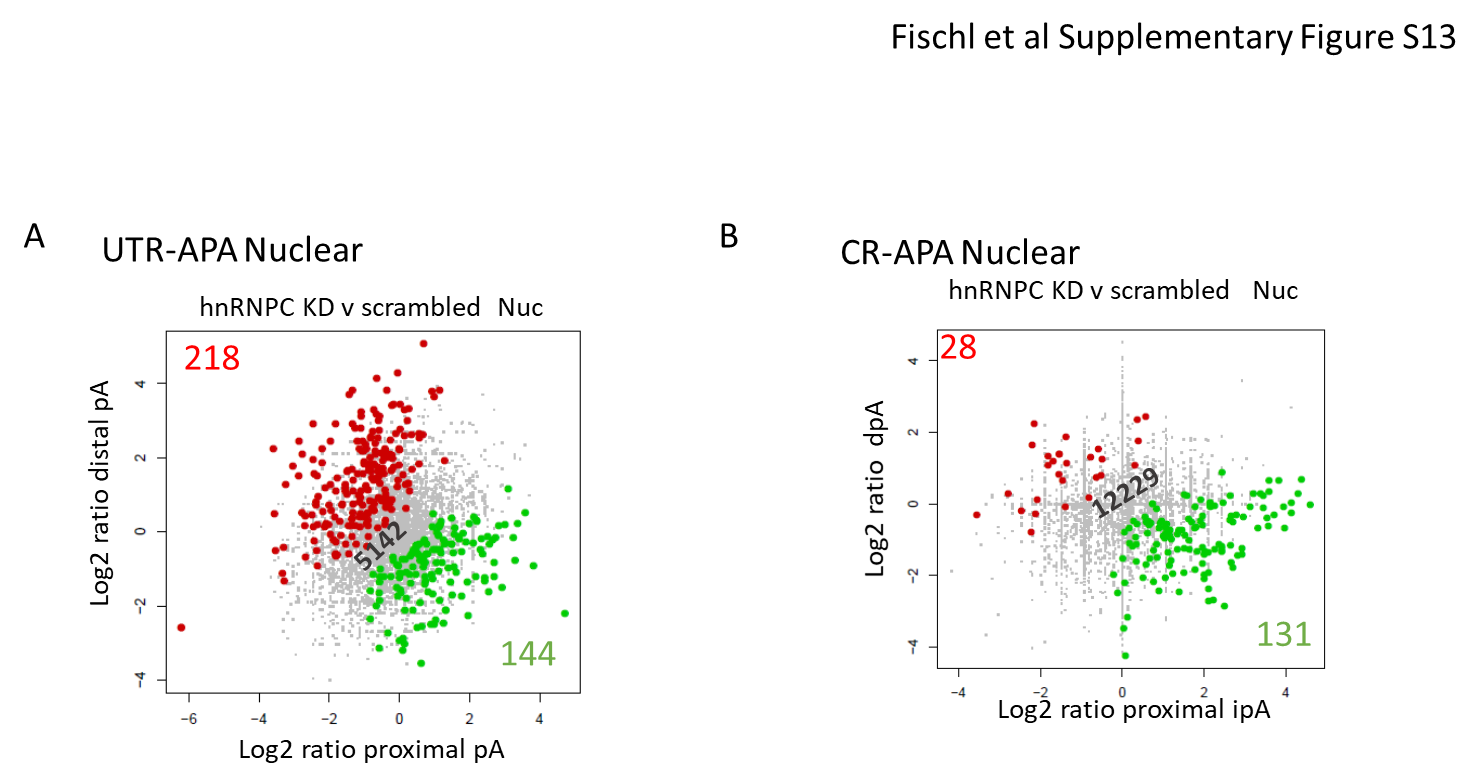


**Supplementary Figure S13. Scatter plots comparing UTR-APA and CR-APA of the nuclear fraction of SW620 cells with normal and reduced hnRNPC levels** (combined replicates)**.**

**A)** Genes with UTR-APA isoforms that differ significantly in relative representation of their most highly expressed UTR-APA isoforms in the nucleus of SW620 cells that have been treated with a scrambled control siRNA (scrambled Nuc) compared to SW620 cells that have been treated with a an siRNA targeting hnRNPC (hnRNPC KD).

**B)** Genes with CR-APA isoforms that differ significantly in relative representation of their proximal (intronic and exonic) APA isoforms relative to the 3’most exon (distal) isoforms in the NUCLEUS of SW620 cells that have been treated with a scrambled control siRNA (scrambled Nuc) compared to SW620 cells that have been treated with a siRNA targeting hnRNPC (hnRNPC KD).

**A) & B)** Grey dots represent genes with UTR-APA or CR-APA isoforms that do not significantly differ in frequency in the nuclear fractions of cells with wild type hnRNPC levels compared to cells that have reduced levels of hnRNPC (hnRNPC KD) between the cell lines. The number of genes undergoing relative shortening (green) or lengthening (red) or genes where isoforms do not change in frequency (grey) are indicated in the bottom right and top left corners and in the centre respectively.


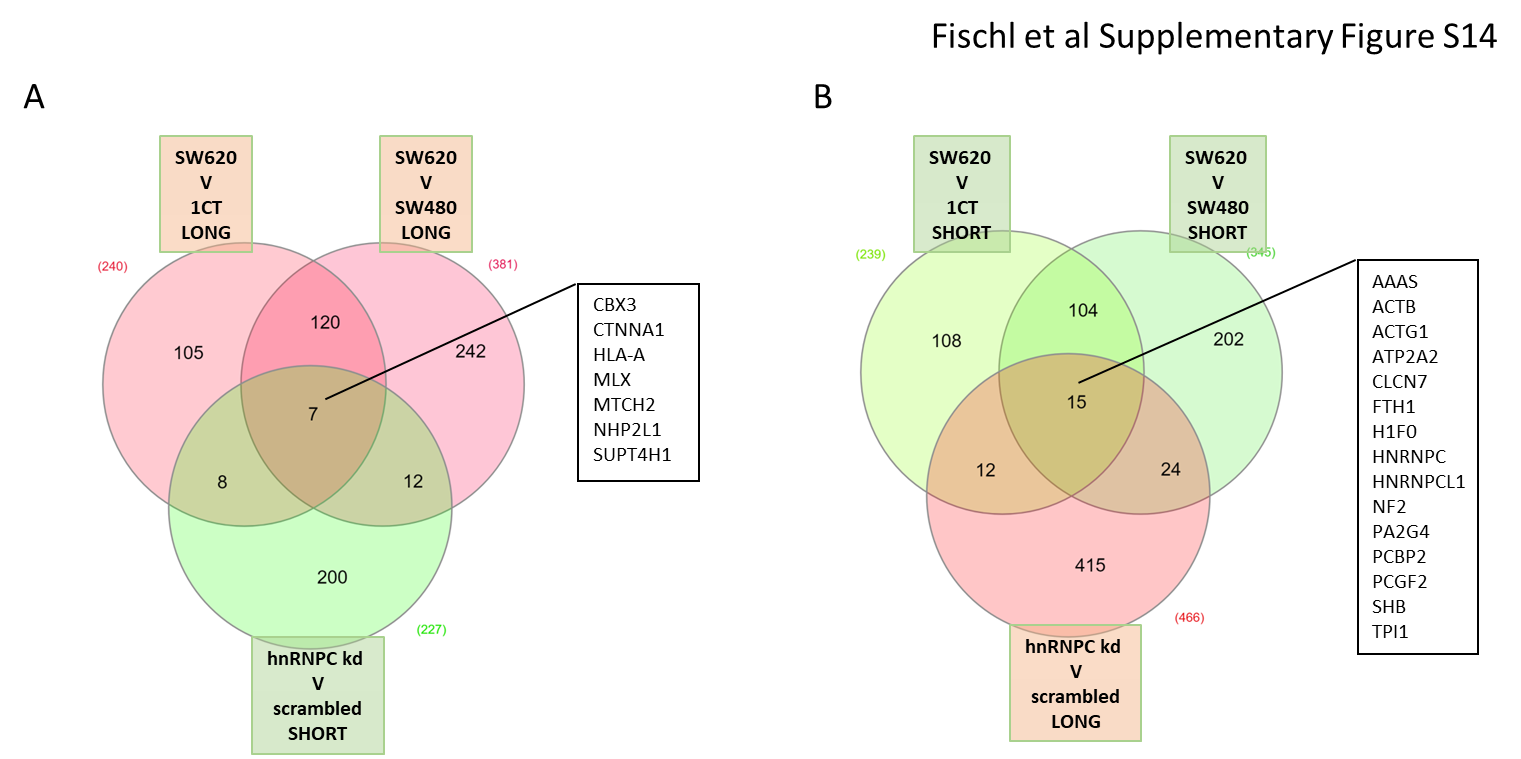


**Supplementary Figure S14. A)** Venn diagram of cytoplasmic mRNA UTR-APA isoforms that lengthen in both the SW620 to 1CT and the SW620 to SW480 comparison and overlap genes that move in the opposite direction (shorten) when SW620 cells are transfected with siRNAs targeting hnRNPC compared to SW620 cells that are exposed to scrambled siRNA treatment. **B)** Venn diagram of cytoplasmic mRNA isoforms that shorten in both the SW620 to 1CT and the SW620 to SW480 comparison and overlap genes that move in the opposite direction (lengthen) when SW620 cells are transfected with siRNAs targeting hnRNPC (hnRNPC kd) compared to SW620 cells that are exposed to scrambled siRNA treatment. Genes that overlap in all three cohorts are listed in the respective boxes.


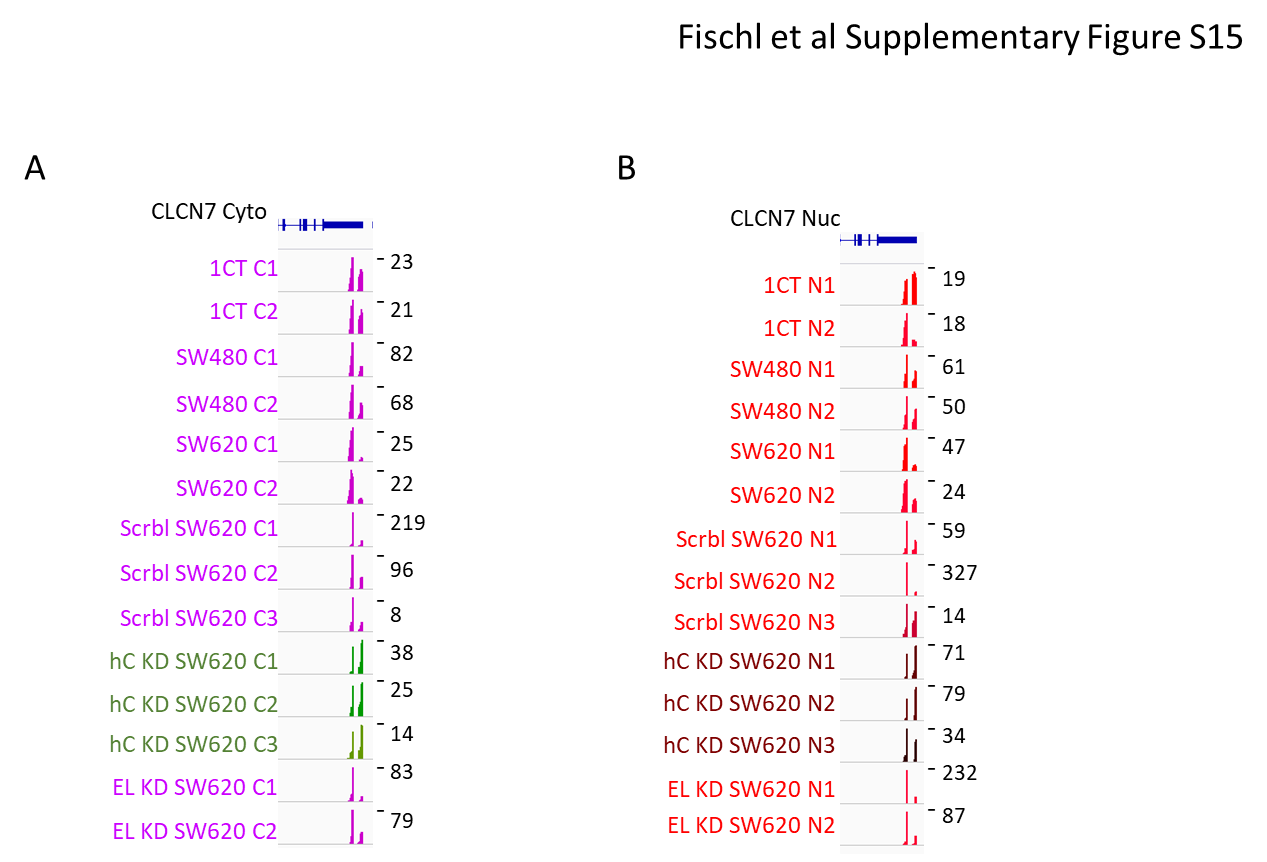


**Supplementary Figure S15. additional example of a gene reverting to 1CT and SW480 status upon hnRNPC KD. UTR-APA changes in CLCN7 upon hnRNPC knockdown in SW620 cells.** hnRNPC knock down reverts nuclear and cytoplasmic CLCN7 SW620 UTR-APA profiles to those, characteristic for SW480 and 1CT cells. **A&B**) Summary of cytoplasmic (A) and nuclear (B) UTR-APA profile changes when hnRNPC is depleted (hC KD SW620) in SW620 cells (green cytoplasm / brown nuclear). Controls are mock scrambled siRNA treated SW620 cells (Scrbl SW620 C1, C2, C3 for cytoplasmic repeats and Scrbl SW620 N1, N2 and N3 for nuclear repeats) and SW620 with reduced levels of ELAVL1 (EL KD SW620 C1/C2) lanes for cytoplasmic and ELAVL1 (EL KD SW620 N1/N2) for nuclear repeats. Read numbers in reads/million are indicated on the right of each lane.


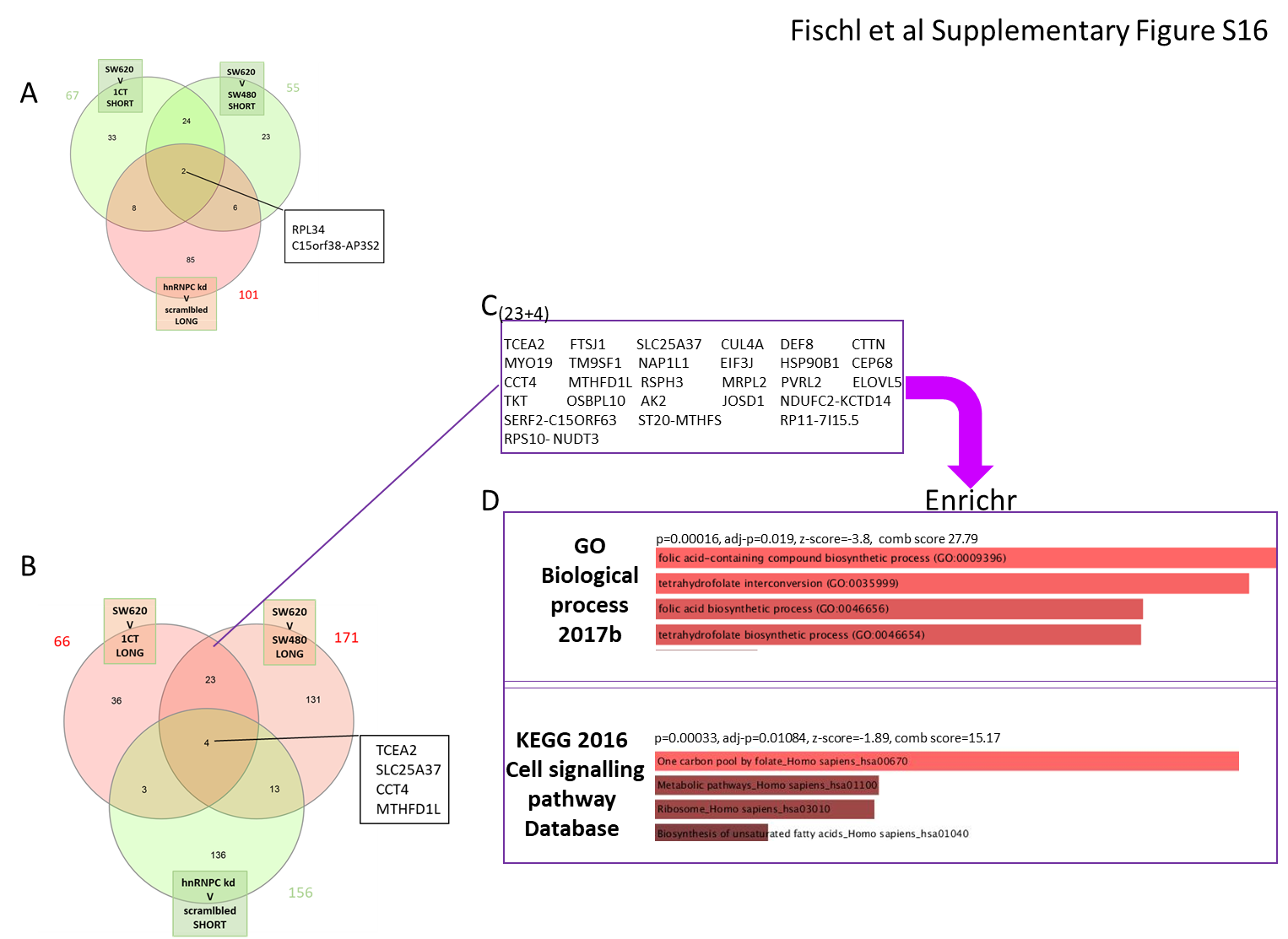


**Supplementary Figure S16.** **A)** Venn diagram of cytoplasmic CR-APA mRNA isoforms that shorten in both the SW620 to 1CT and the SW620 to SW480 comparison and overlap genes that move in the opposite direction (lengthen) when SW620 cells are transfected with siRNAs targeting hnRNPC compared to SW620 cells that are exposed to scrambled siRNA treatment. Genes that overlap in all three cohorts are listed in the text box to the right. **B)** Venn diagram of cytoplasmic mRNA CR-APA isoforms that lengthen in both the SW620 to 1CT and the SW620 to SW480 comparison and overlap genes that move in the opposite direction (shorten) when SW620 cells are transfected with siRNAs targeting hnRNPC compared to SW620 cells that are exposed to scrambled siRNA treatment. **C)** List of the genes that show a higher frequency of the full-length CR-APA isoforms compared to the short CR-APA isoform caused by usage of an internal poly(A) site in both the SW620 v 1CT and SW620 v SW480 comparisons. **E)** Enrichment analysis of the list shown in “C)” using the “Enrichr” platform (Chen et al. *BMC Bioinformatics* 2013; 14:128; Kuleshov et al. Nucleic Acids Res, 2016, 44(W):W90-7)) The top four hits are shown and p values, adjusted p values, z-scores and combined scores of the top hit in the two databases are indicated above the bar graphs.


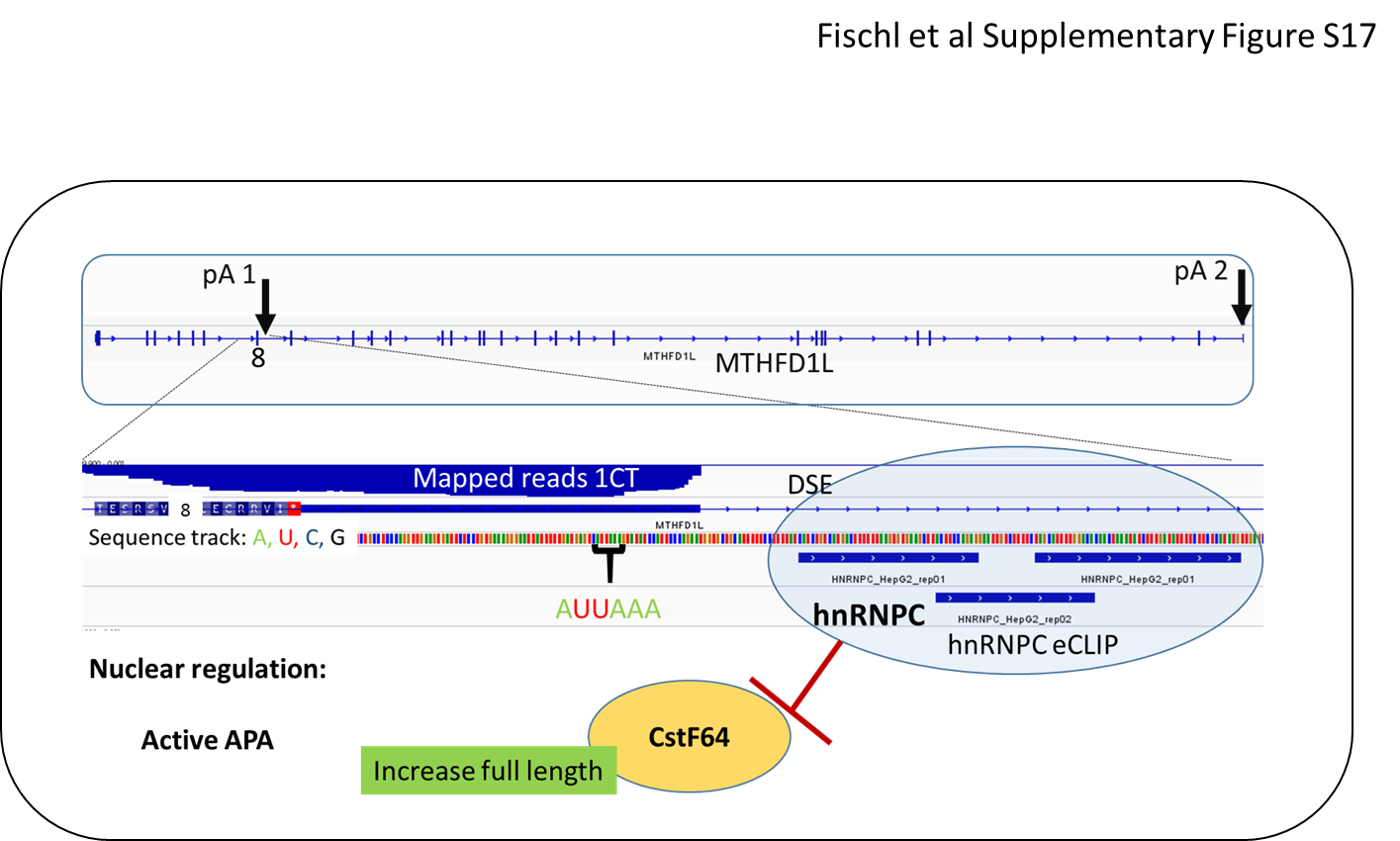


**Supplementary Figure S17**: **Proposed model how hnRNPC could modulate APA of MTHFD1L.** High levels of hnRNPC such as observed in SW620 cells may impair the recognition of the downstream sequence element (DSE, uridine rich region) of the internal poly(A) site (pA1) by the cleavage and polyadenylation stimulatory factor (CstF64) by binding to hnRNPC binding site that overlap/flank the DSE region (hnRNPC eCLIP tracks (ENCODE; [GEO:GSE91860](https://www.ncbi.nlm.nih.gov/geo/query/acc.cgi?acc=GSE91860)). This will cause suppression of pA1 and force read through at pA1 in the nucleus resulting in an increased usage of pA2. This APA event shifts the balance from short to full- length MTHFD1L mRNA isoforms. The MTHFD1L gene structure with the two poly(A) site sites “pA1” and “pA2” is shown in the central box and below is a zoomed in view of the region surrounding the internal poly (A) site pA1 with reads mapped to the end of exon 8 (8) in blue. The nucleotide composition of this region is indicated by the green, red, blue and black coloured vertical lines. The hnRNPC eCLIP data are shown as blue bold lines with white dots and the DSE and the AUUAAA hexamer of the internal polyA site are indicated. The mapped reads representing the sequenced 3' ends of extracted RNA from the 1CT cytoplasmic fractions from repeat1 of the 1CT cells are indicated in bigwig format above the zoomed in gene structure for the pA1 or pA2 regions.


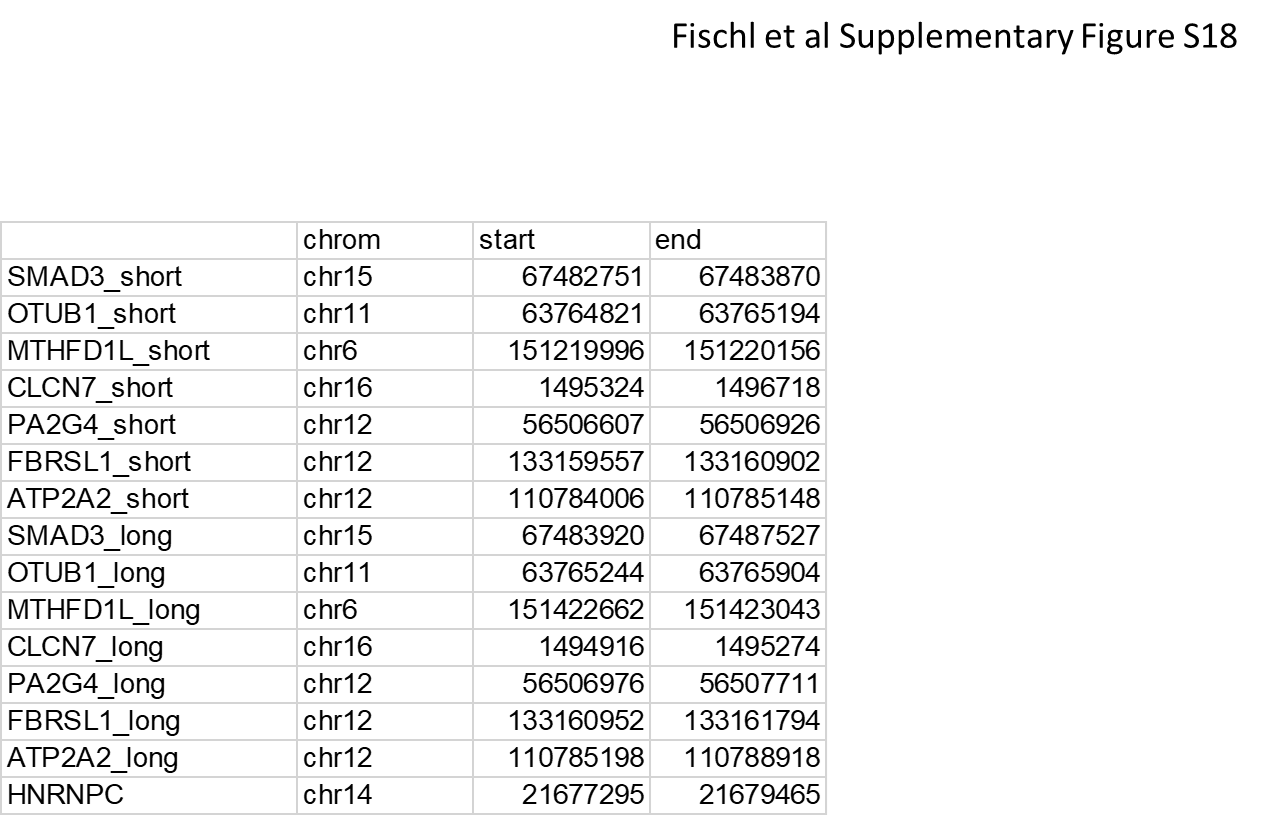


**Supplementary Figure S18.** Table of counting regions used for each gene to calculate gene expression data and ratios of APA isoforms as shown in Figure 6 and Figure S7.


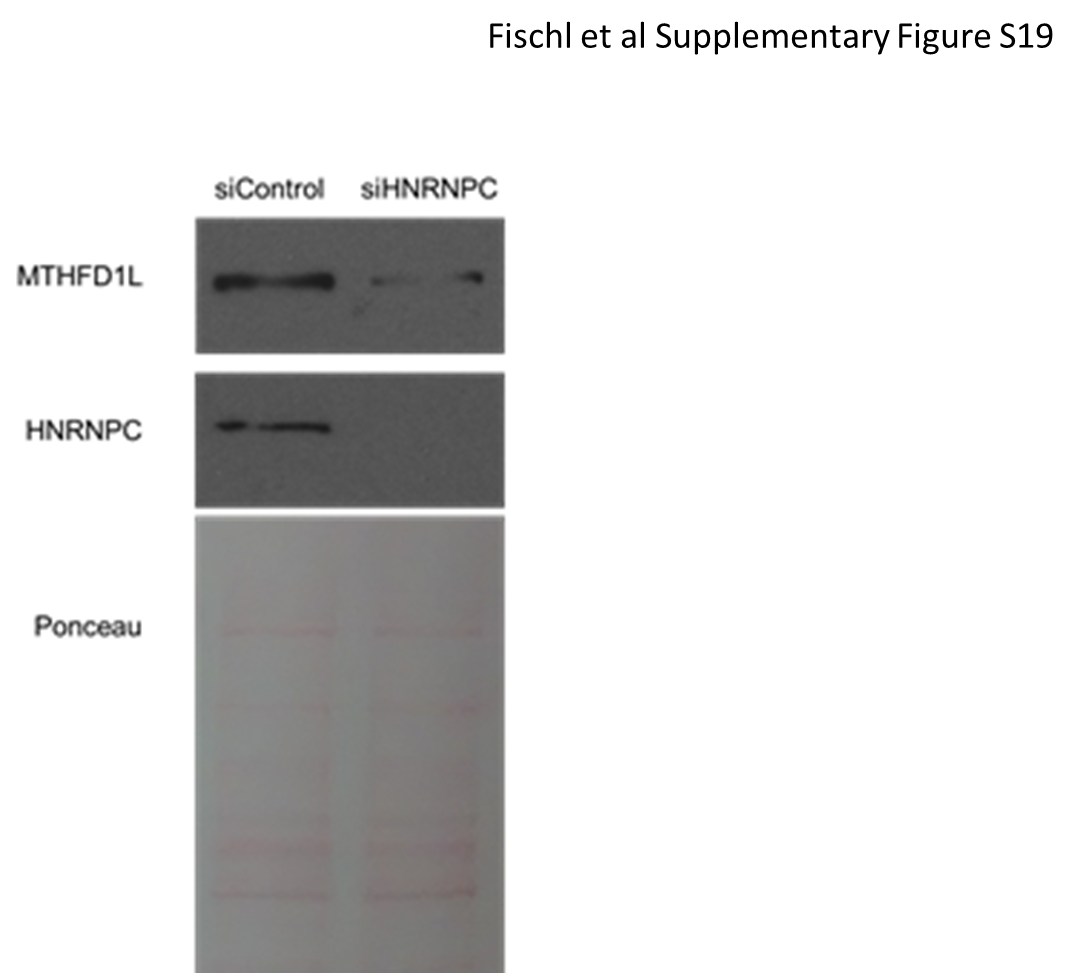


**Supplementary Figure S19.** Western blot of total protein extracts targeting full-length *MTHFD1L* (top panel) and hnRNPC (middle panel) in SW620 cells with reduced levels of hnRNPC compared to control cells that have been transfected with an siRNA targeting ELAVL1. Ponceau stain (bottom panel) shows equal loading.


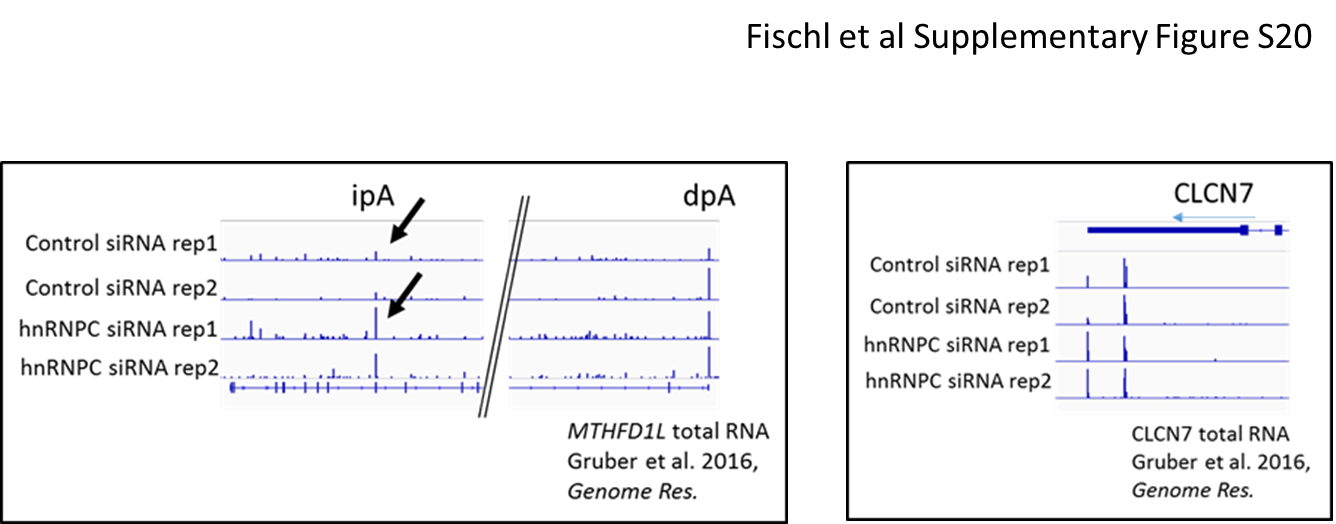


**Supplementary Figure S20.** Genome browser views of MTHFD1L (left panel) and CLCN7 (right panel) comparing control siRNA treated Hek293 cells with those transfected with siRNAs targeting hnRNPC. The bigwig files were obtained from Gruber et al. 2016; Genome Res.; 8:1145-59.


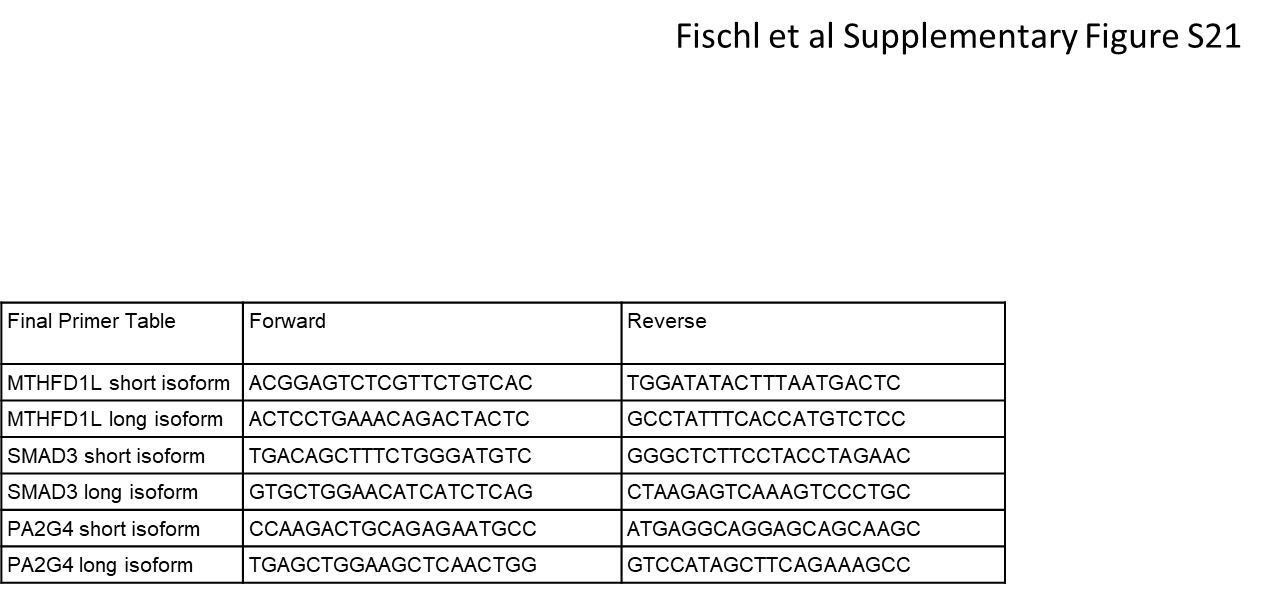


**Supplementary Figure S21.** List of primers used for the RT-PCR analysis of the MTHFD1L, SMAD3 and PA2G4 APA isoforms.
